# Climate seasonality and predictability during the middle stone age and implications for technological diversification in early *Homo sapiens*

**DOI:** 10.1038/s41598-025-95573-y

**Published:** 2025-04-04

**Authors:** Lucy Timbrell, James Clark, Gonzalo Linares-Matás, Solène Boisard, Eslem Ben Arous, James Blinkhorn, Matt Grove, Eleanor M. L. Scerri

**Affiliations:** 1 Human Palaeosystems Group, Max Planck Institute of Geoanthropology, Jena, Germany; 2 Department of Archaeology, Classics and Egyptology, University of Liverpool, Liverpool, UK; 3 Corpus Christi College, University of Cambridge, Cambridge, UK; 4 Emmanuel College, University of Cambridge, Cambridge, UK; 5 Department of Anthropology, University of Montreal, Montreal, QC Canada; 6 Centro Nacional de Investigación sobre la Evolución Humana (CENIEH), Burgos, Spain; 7 Muséum national d’Histoire naturelle, Histoire Naturelle des Humanités Préhistoriques, CNRS- MNHN-UPVD, Paris, France; 8 Department of Classics and Archaeology, University of Malta, Valletta, Malta; 9 Department of Prehistoric Archaeology, University of Cologne, Cologne, Germany

**Keywords:** Human-environment interaction, African archaeological record, Hunter-gatherer adaptations, Aterian, Palaeoclimatic change, Archaeology, Cultural evolution, Evolutionary theory, Evolutionary ecology, Palaeoecology

## Abstract

Regionalisation is considered to be a hallmark of the Middle Stone Age (MSA) compared to the Early Stone Age. Yet what drove diversification around a shared technological substrate that persisted across Africa for hundreds of thousands of years remains debated. Non-mutually exclusive hypotheses include region-specific styles in manufacture, social signalling, cultural drift between geographically isolated populations, and diverse environmental adaptations, as well as the impacts of unequal research histories and intensities. We explore the potential ecological bases of behavioural diversity during the MSA between two well-studied and diverse areas: eastern and northwestern Africa. We utilise a set of standardised bioclimatic simulations, as well as a time series decomposition algorithm, to determine the nature and extent of regional differences in terms of environmental productivity, seasonality and predictability at MSA sites through time. Our results highlight that, compared to human occupations of eastern Africa, northwestern African MSA occupations are associated with colder, drier and less productive environments, albeit colder, but wetter and more productive compared to surrounding areas, with higher temperature seasonality and more predictable climates across millennia. We then theoretically consider the implications of our results for technological diversification between these two regions during the Middle to Late Pleistocene, such as for the investment in specific risk mitigation strategies for dealing with seasonally mobile resources in northern localities, and the diversification of MSA toolkits in tropical eastern Africa.

**Supplementary Information:**

The online version contains supplementary material available at 10.1038/s41598-025-95573-y.

## Introduction

The Middle Stone Age (MSA) represents the earliest behavioural signature of our species, *Homo sapiens*, across Africa from ca. 300,000 to 30,000 years ago (ka)^1–3^.The early MSA is denoted by the consistent appearance of prepared-core and flake stone technology and the use of standardised pointed pieces^4^, but the MSA is also notable for its later regional diversity, with clearly identifiable elements appearing in some but not all areas of Africa^5–7^. Such regional innovations include localised hunting and processing technologies on both stone and bone^8,9^, such as bow-and-arrow use, as well as personal shell beads^10,11^, and the engraving of objects^4,6,8,12^. For this reason, the MSA in certain areas (e.g., parts of northern and southern Africa) has historically been divided into actual or perceived cultural turnovers, whereas the archaeological records in equatorial Africa tend to be emphasised as highly variable without such (relatively) clear spatiotemporal boundaries in material culture^7,13^.

Many hypotheses have been put forward to explain these differences in the emergence and diversification of the MSA. Some have suggested that increased climate variability during the Middle Pleistocene led to the onset of ‘generalist’ MSA behaviours, contributing to the increased ecological and technological flexibility of *Homo sapiens*^14–16^. Others have pointed to cognitive differences between early modern populations^17^, including in specific cognitive domains that are shaped by local environmental and phylogenetic pressures^18^. However, a multitude of ecological, demographic and social factors, as well as their interplay, were possibly responsible for the differential adoption of certain MSA traits in different regions of Africa across time^6,7,19^. This model better accounts for the loss of distinctive innovations in certain regions at times of pressure exerted by these different factors^20^. Recent analyses support a hierarchy of interlinked influences on modern hunter-gatherer behaviour, where variation in the toolkit at the most proximate level is driven by the type of resources being consumed, which in turn shapes the size and structure of the population that can be supported in a given area^21,22^. Most clearly this is linked to fluctuations in the availability of plant resources, as these foodstuffs can be exploited with the ‘simplest’ tool forms^22^, defined by Oswalt^21^ as those with the fewest number of individual components. Conversely, hunter-gatherers occupying areas with reduced plant availability tend to require higher fish and meat consumption, which are associated with more complex (i.e., modular) tools, decreased population density, and greater maximum seasonal settlement sizes—at the cost of additional energetic investment^21,22^.

In this context, numerous authors have emphasised the importance of “ecological risk” in governing behavioural investment at large^21,23–25^, whereby selective pressures favouring specific technological adaptations only become apparent where there is no viable alternative^22^. At the same time, researchers disagree on how to operationalise the nature of ecological risk for hunter-gatherer populations. For Oswalt^21^, risk is related to the mobility of the resources being targeted and their respective media (i.e. land vs. water), because the probability of capture strongly declines for highly mobile, and especially aquatic, resources. Torrence^23^ argues that, while the speed and success of resource capture is the proximate driver of technological composition, risk is an overarching concept defined by the probability of not meeting overall dietary requirements and the related cost of such a failure. Collard et al.^24^ take latitude and effective temperature as proxies for risk, while Thompson et al.^25^ tie risk in the African context to reduced precipitation and general water availability. All of these non-independent metrics are likely contributors to ecological risk across different geographic and temporal scales, as well as environmental contexts, with diverse implications for technological variability for hunter-gatherers during the MSA. For example, behavioural adaptation in inter-tropical environments with high plant availability is more likely to be driven by rainfall and its seasonality^26,27^, whereas fluctuations in temperature are more important for understanding changes to hunting and fishing patterns amongst groups that occupy temperate and arctic zones^22^.

On the other hand, technological variability within individual foraging strategies is likely driven by demographic structure and how it relates to the total amount of environmental and cultural information that can be sustained by a population. For example, higher encounter rates between populations, greater population density, and/or increased raw population sizes have been hypothesised to provide the capacity for innovation through increased cultural transmission and sharing, and the population-level distribution of technological know-how^28–31^. Clark and Linares-Matás^32,33^ have previously argued that increases in such “landscape knowledge” are critical for further technological investment because they govern the predictability of corresponding returns. This allows individuals and groups to decide whether returns are high and consistent enough for behavioural adaptation to be worthwhile.

However, there is no reason to think of these different processes as mutually exclusive. Rather, they act in different ways, at different scales, and on different elements of the behavioural system^7,22,30^. Both ecological risk and demographic structure are also subject to change through time; alongside broader shifts in climate, these are crucial for linking ecology to evolutionary processes^34^. Variation in the density and the spatio-temporal availability of resources is associated with the development and investment in different technological and demographic risk-management systems, to mitigate fluctuation in the variance and overall returns of foraging^33^. With regards to technology, specifically, increased unpredictability in resource distributions between years constrains the amount of landscape knowledge that can be accumulated within the population, and therefore the amount of investment that can be put into the toolkit^33^. Instead, technological strategies in these circumstances should be focused on ‘generalised’ toolkits that can be used in several different tasks with suboptimal efficiency, following key principles from evolutionary biology regarding the links between (phenotypic) plasticity and temporal climatic variability^35–37^. It is only within predictable environments that enough landscape knowledge can be accumulated within each foraging domain to invest in specialised tools for highly specific tasks^33^. High tool-to-task fit is explicitly linked to technological complexity, as specific tools tend to be highly curated in order to perform well in certain contexts, often resulting in more elaborate technologies in ethnographic societies^22,32,33^.

We use this theoretical framework as a lens through which to explore differences in the potential ecological bases of technological diversity between different regions of the African MSA. As a large and ecologically variable continent, rates and the extents of change in temperature, precipitation, and net primary productivity (NPP) are experienced differently in different areas of Africa, with some regions showing greater levels of diversity through space and time. Populations inhabiting tropical Africa, for example, may be more insulated against the extremes of change compared to those at more extreme latitudes where glacial periods would have been felt relatively harsher^38,39^, with localised dispersals along steep altitudinal gradients during periods of climatic fluctuation in eastern Africa^40,41^. In the same way, the Atlantic and Mediterranean coast of northwestern Africa may have acted as a refugium throughout the last ca. 100 ka for human populations^42^ and some floral and micromammal species^43–45^, thanks to buffer effects of the ocean and mountains. Furthermore, asynchronous climate responses to orbital forcing and the impacts of the Walker Circulation in different latitudinal and longitudinal zones created a mosaic of habitat shifts within the continent through time^38,46^. As such, we should not expect identical behavioural responses to the environment in different regions of Africa throughout the MSA, because the mechanisms involved are numerous and their relationships still poorly understood.

To this end, here we explore the different ecological correlates of MSA sites between northwestern (*n* = 21) and eastern Africa (*n* = 37), specifically focusing on the extent and nature of seasonality and climate predictability in these environments from ca. 332–25 ka. We have selected these regions because:

i) they represent an ideal test case for understanding drivers of technological change throughout the MSA, given their very distinct ecologies^36^.
ii) they also remain two of the most well-studied areas for MSA populations in the continent^7,47^, with comparable datasets of dated phases of human occupation available for synthetic analysis^48,49^.
iii) they are thought to show different expressions of MSA technology (see Timbrell^7^ and Ben Arous et al.^47^for recent reviews), with the northern African record typically divided into distinctive groups of assemblages (e.g. Aterian, Mousterian, Nubian) based on non-homogenous but specific elements (though not without critique^50^). The eastern African record contrastingly is thought to feature a complex mosaic of (often site-specific) industrial sequences, with no single overarching regional culture-historical framework for the MSA due to this marked variability^51^. We provide a brief overview of the archaeological records in each region in SOM 1.

**Fig. 1:**
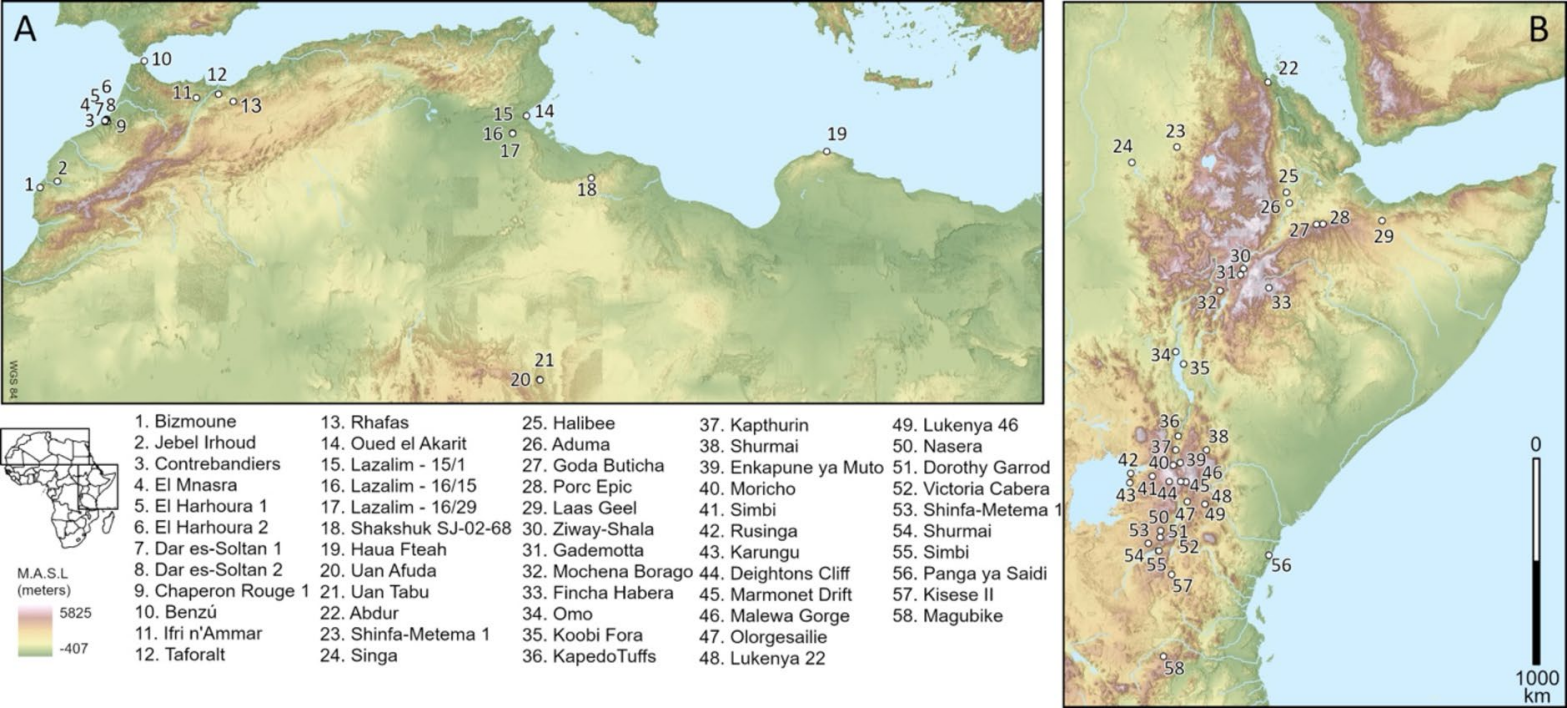
Geographic distribution of the northwestern (**A**) and eastern (**B**) African MSA site locations studied in this research^48,49^.

This study does not seek to validate or invalidate MSA taxonomic classificatory systems but rather explore ecological differences between regions to determine what may have constituted risk and how this could relate to technological diversification and investment more broadly during the MSA across Africa. We deploy these existing datasets of MSA sites in the two regions across its entire range (Fig. 1) and generate comprehensive climatic information for each site during times of hominin occupation using established model simulations of standardised bioclimatic variables^52^and a time series decomposition algorithm^53^.

## Results

### Mean annual climate parameters

**Fig. 2:**
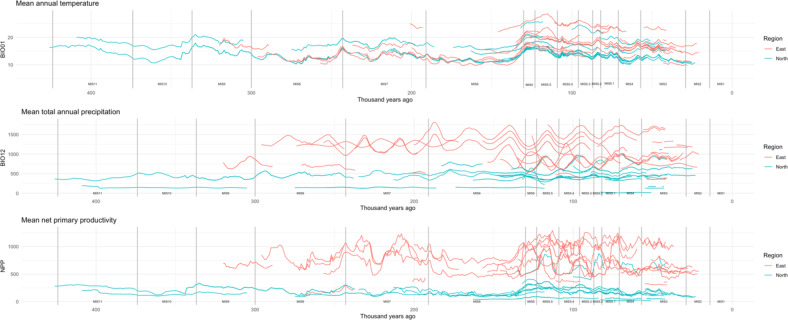
Time series of mean annual temperature (bio01; °C) and total annual precipitation (bio12; mm) and net primary productivity (NPP) across the dating range of all Middle Stone Age occupations in eastern (red) and northwestern (blue) Africa. Marine Isotope Stages are denoted, based on Lisiecki and Raymo^80^.

We first compared mean annual temperature (bio01), mean total annual precipitation (bio12) and mean NPP across unique MSA occupations of archaeological sites in eastern (*n* = 59) and northwestern (*n* = 106) Africa (Supplementary Table S1). Figure 2 highlights time series for these three climate variables from the minimum to maximum date of all dated occupations. Notably, MSA occupations in northwestern Africa tend to be associated with significantly lower precipitation, temperature and NPP compared to eastern African occupations (all *p* < 0.001). These comparisons using the mid-age estimates were found to be robust in sensitivity analyses for all three variables, returning significant results in all 1000 permutations across the date ranges of the occupations (Supplementary Table S2 and Figure S1).

Within eastern Africa, there is significantly more intra-regional variation compared to northwestern Africa across all three variables (all *p* < 0.05), which is consistent across the 1000 iterations permuted across the date range for precipitation and NPP (Supplementary Table S3 and Figure S2). Results for temperature are more inconsistent, with only 329 iterations similarly producing significant results and the test coefficient using the mid-age values falling within the 16th percentile of the permuted distribution (Supplementary Table S3 and Figure S2). Comparisons using the coefficient of variation shows that, whilst eastern Africa is confirmed as having more variable annual temperatures (eastern Africa = 22.8%, northwesterern Africa = 15.07%), occupations within northwestern Africa have slightly more varied annual precipitation and NPP when taking into account that these parameters are on average significantly lower in this region (bio12: eastern Africa = 30.5%, northwestern Africa = 35.2%, NPP: eastern Africa = 32.56%, northwestern Africa = 46.78%).

When comparing annual climates at MSA occupations against the regional background via random sampling, we found that in almost every iteration, statistically significant differences are observed (in eastern Africa: bio01 = 990/1000, bio12 = 964/1000, NPP = 998/1000, in northwestern Africa: all iterations). In both regions, MSA occupations tend to be in colder, wetter and more productive environments compared to random background samples through both time and space (Supplementary Figure S3). However, in eastern Africa, the mean climatic values observed at MSA occupations (based on the mid-age) fall within the distributions of the random samples (Supplementary Figure S3) albeit at the extremes (leading to statistically significant differences in almost all cases), yet for northwestern Africa, the climatic conditions at MSA occupations fall considerably beyond that produced by random sampling, confirming that environmental conditions were stronger mediator of the spatiotemporal patterning in human occupation in northwestern Africa (Supplementary Figure S3).

### Temperature and precipitation seasonality

**Fig. 3:**
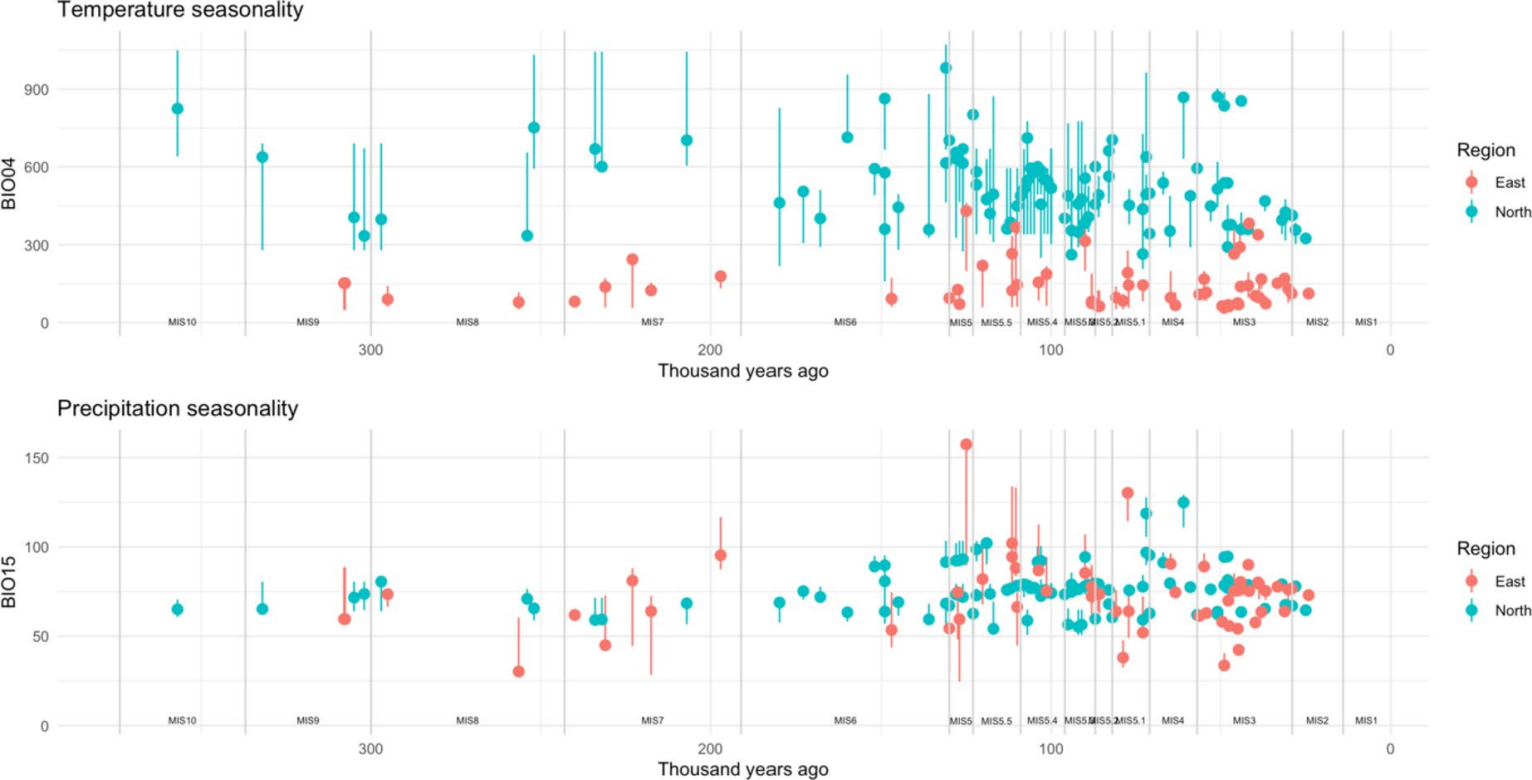
Temperature (bio04; °C) and precipitation (bio15; mm) seasonality experienced during Middle Stone Age occupations in eastern (red) and northwestern (blue) Africa at the mid-age (circles) and across the date range (lines). Marine Isotope Stages are denoted, based on Lisiecki and Raymo^80^.

We next explored temperature and precipitation seasonality across MSA occupations in northwestern and eastern Africa (Fig. 3). Using mid-age values, occupations in eastern Africa tend to have significantly lower temperature seasonality compared to northwestern African occupations (*p* < 0.001), whereas precipitation seasonality is not significantly different between the regions (*p* = 0.091). Sensitivity analyses found these results to be robust when permuting climatic values extracted from across the date range; all 1000 iterations returned significant results for temperature seasonality whereas 947 similarly returned non-significant results for precipitation seasonality, with the test coefficients using the mid-age falling into the centre of the permuted distributions, (Supplementary Table S2 and Figure S1).

Occupations within northwestern Africa have significantly higher variance in temperature seasonality compared to eastern Africa (*p* < 0.001) whereas variance in precipitation seasonality is not significantly different (*p* = 0.06), with all 1000 iterations returning significant results for temperature seasonality and 969 for precipitation seasonality (Supplementary Table S3 and Figure S2). However, the test coefficient for precipitation seasonality suggests that the mid-age estimates produce results in the 98th percentile of the distribution, highlighting potential unreliability (Supplementary Table S3 and Figure S2). Nonetheless, calculation of the coefficient of variation also supports that northwestern African occupations show less intra-regional variability in seasonality, particularly in terms of temperature (bio04: eastern Africa = 60.19%, northwestern Africa = 28.72%, bio15: eastern Africa = 28.15%, northwestern Africa = 16.52%). Almost all of the random samples of the regional background were significantly different from the mid-age seasonality estimates (bio04 in eastern Africa = 977/1000 iterations, bio15 in eastern Africa = 996/1000 iterations), with MSA occupations tending to be in less seasonal areas within each region (Supplementary Figure S3).

There appear to be few clear chronological trends in seasonality occupied by MSA populations in each region in terms of the mid-age (marked as circles in Fig. 3). Variability in precipitation seasonality across the date-range is limited in northwestern African occupations compared to those in eastern Africa, even in occupations with wider dating uncertainty associated (and so more timeslices extracted). Standardising the variance by dating uncertainty at each occupation supports this trend (Supplementary Figure S4). For the more numerous occupations during MIS 5, there is strong intra-occupation diversity in temperature seasonality in northwestern Africa, whereas during MIS 3 in eastern Africa there is diversity in precipitation seasonality across the date range (Fig. 3), though this is reduced considerably when standardising by age uncertainty (Supplementary Figure S4).

In eastern Africa, occupations of Laas Geel (Somaliland), Abdur (coastal Eritrea), Halibee Farm and Porc Epic (both Ethiopia) are associated with elevated temperature seasonality compared to other sites in this region, with Abdur also experiencing the highest precipitation seasonality of entire dataset based on the mid-age, though there is much variation across the date range (Fig. 3; Supplementary Table S1). Moricho and Enkapune Ya Munto (Central Rift Valley in Kenya), Karungu and Rusinga (both near/in Lake Victoria in Kenya) exhibit the lowest precipitation seasonality in this region. In northwestern Africa, occupations of Oued el Akarit, Wadi Lazalim (both Tunisia), Rhafas S7 (Morocco), Uan Tabu, and Uan Afuda (both Libya) have elevated temperature seasonality compared to other sites in northwestern Africa, the latter two sites also representing the occupations with the highest precipitation seasonality.

### Climate predictability

To calculate inter-millennial predictability, we utilised the change/variability decomposition (CVD) algorithm (see Methods) for differentiating between ‘change’ and ‘variability’ elements in climatic time series^53^. Here, ‘change’ is recognised as the autocorrelated shift in the state of climate across extended timeframes like precession cycles^53^. In contrast, ‘variability’ reflects random fluctuations in a time series once change has been corrected for, acting as a proxy for climate unpredictability^53^. We therefore extract the modelled time series from site locations across dated occupations and calculate the percentage of the signal explained by ‘change’’ versus ‘variability’. Sites where the percentage of variability is higher are predicted as having more unpredictable climates.

**Fig. 4:**
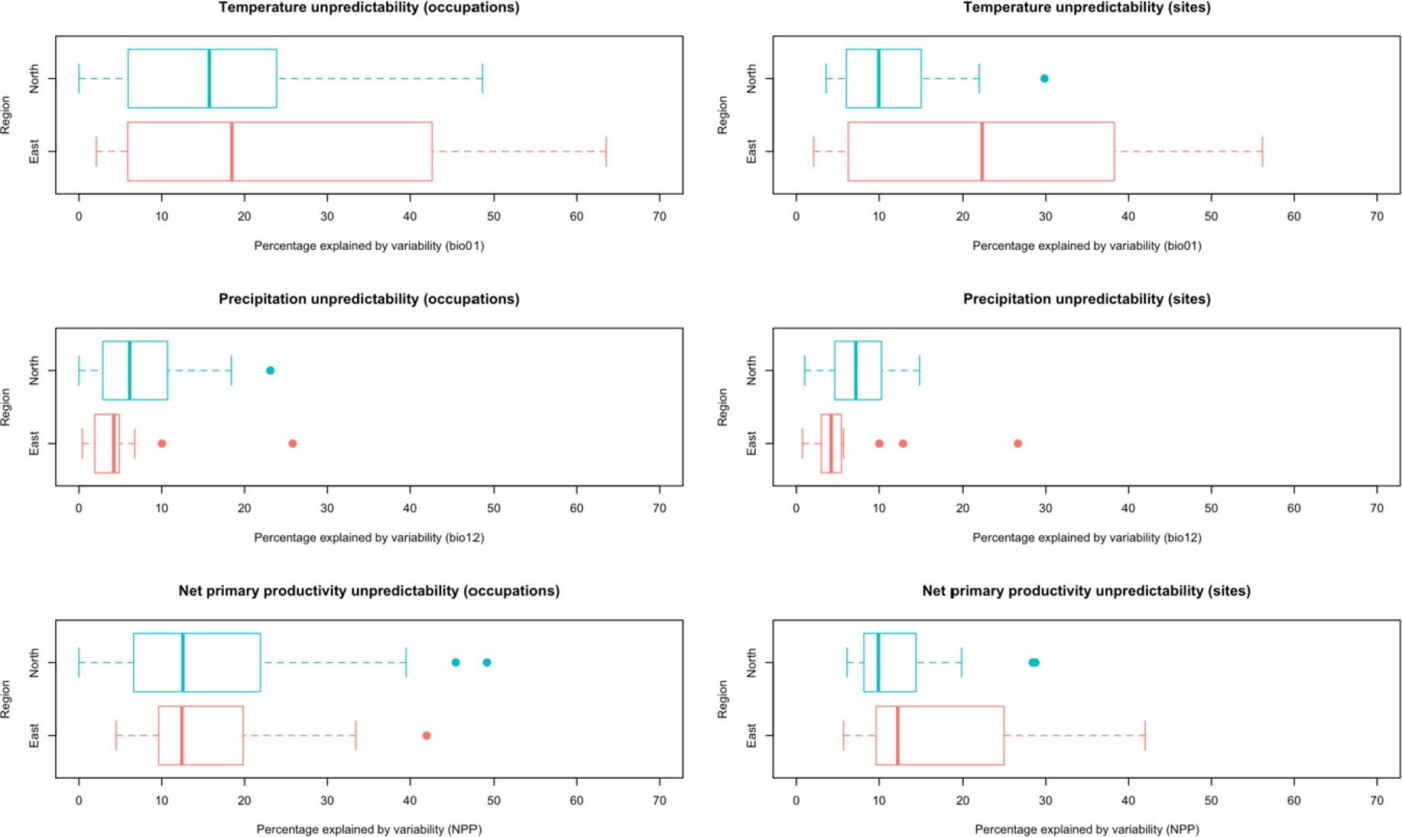
Boxplots of the percentage of variability in mean annual temperature (bio01), total annual precipitation (bio12) and net primary productivity (NPP), comparing 20 eastern African and 37 northwestern African unique occupations (left) and 16 eastern African and 15 from northwestern African sites (right).

We first focussed on the 57 unique MSA occupations (eastern Africa = 20, northwestern Africa = 37) that have date ranges covering a full precession cycle (~ 23,000 years). Supplementary Table S1 reports the percentage of change-corrected variability aspects of mean annual temperature, total annual precipitation and NPP for each of these occupations. In general, mean annual temperature and net primary productivity tend to have larger proportions of variance related to variability (unpredictability) compared to change, whereas for precipitation almost all of the variance relates to change (Fig. 4). Figure 4 demonstrates that, within individual occupations, temperature unpredictability is higher (*p* = 0.197) and more variable (*p* = 0.078) in eastern Africa compared to northwestern Africa, the latter with close to significant differences. Northwestern African occupations have on average less predictable rainfall compared to eastern Africa (*p* = 0.131), with close to significant differences in the percentage of the time series explained by variability (*p* = 0.063) and variability within regions (*p* = 0.059). For NPP, we find no significant differences in the average percentage of the variability component in the time series between the two regions (*p* = 0.861) though the intra-regional variation is significantly greater in northwestern Africa (*p* = 0.027).

**Fig. 5:**
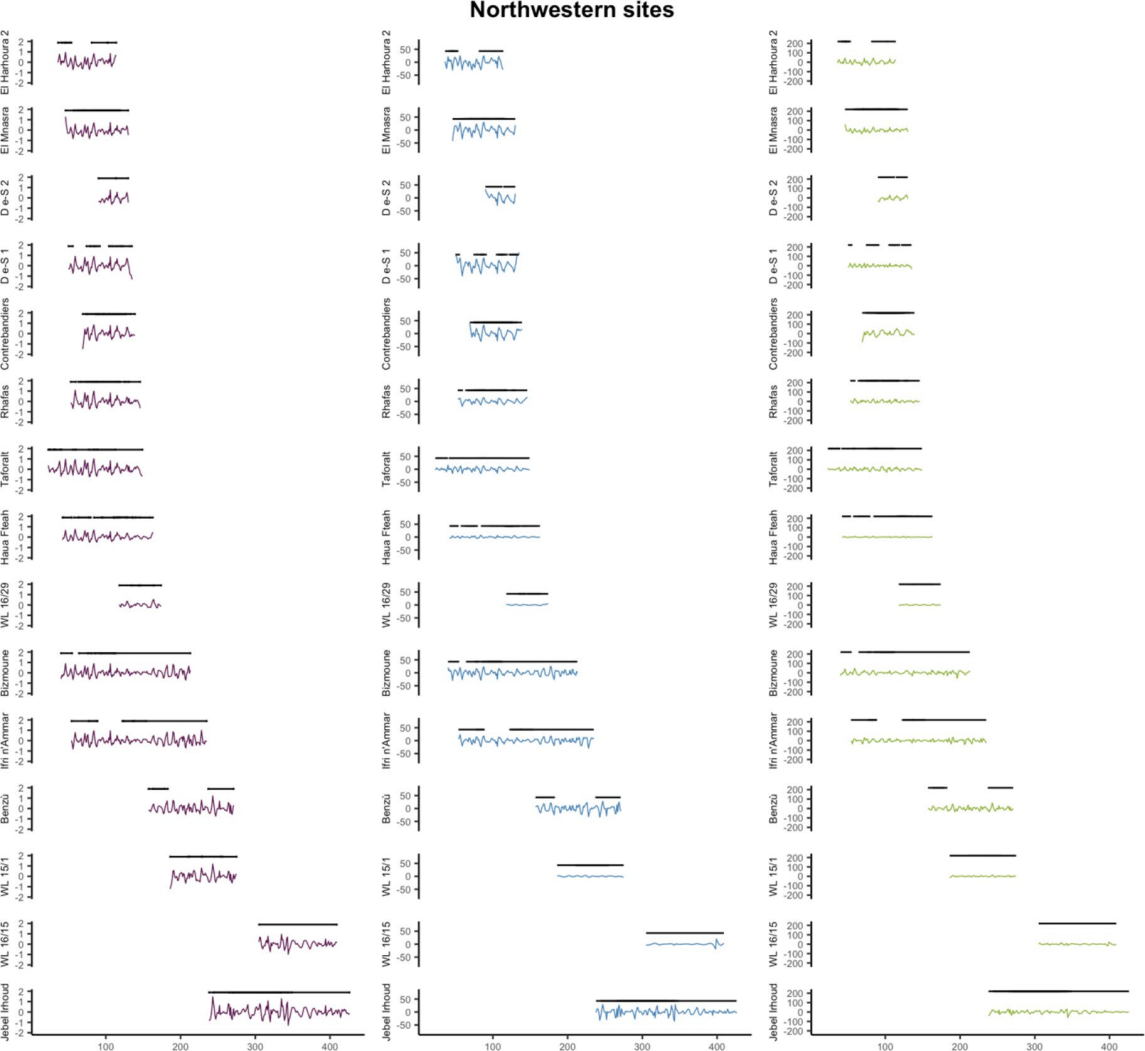
Temperature (left), precipitation (middle) and net primary productivity (right) unpredictability at northwestern African Middle Stone Age sites through time, with date ranges of distinct occupations (black lines).

**Fig. 6:**
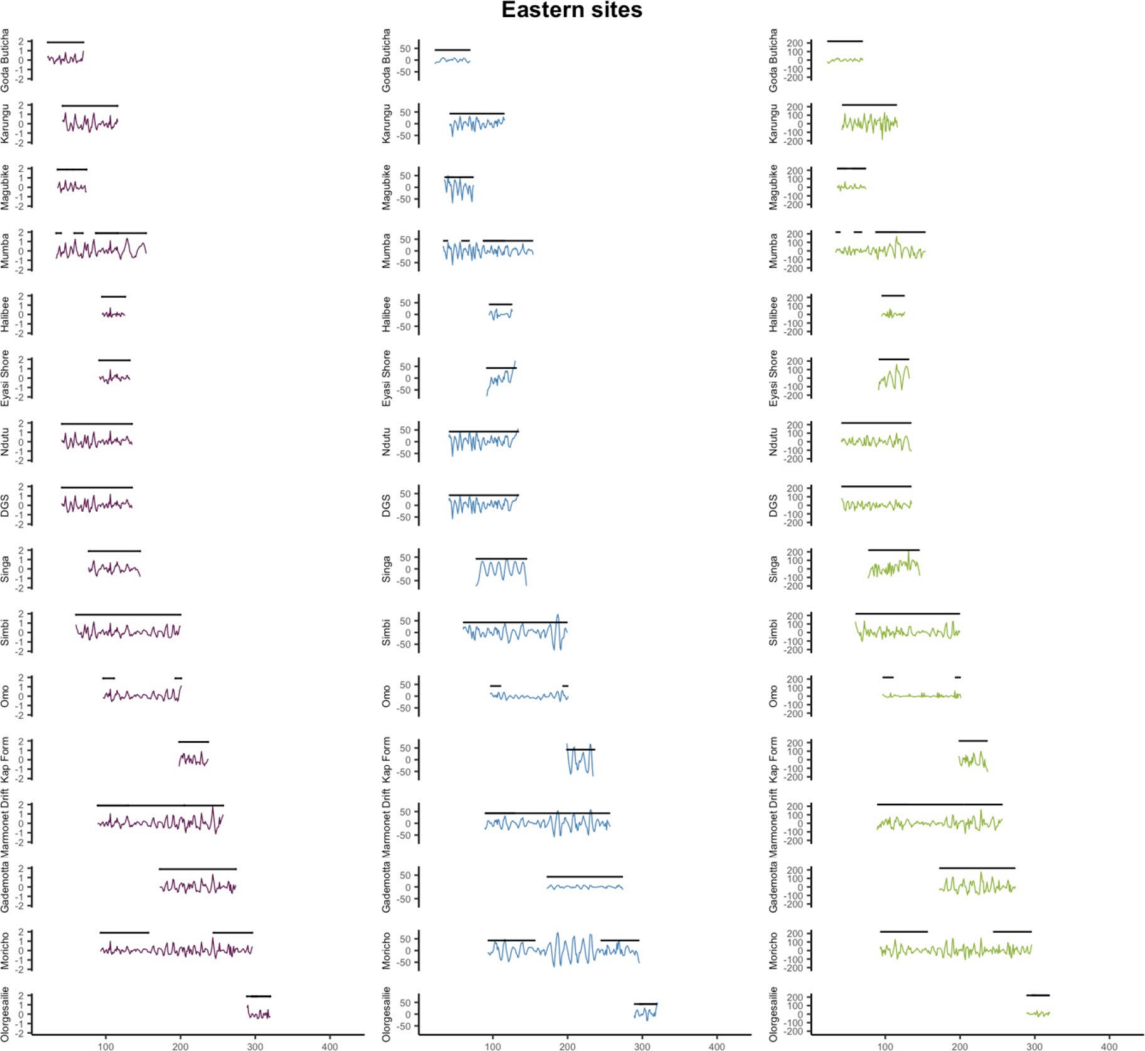
Temperature (left), precipitation (middle) and net primary productivity (NPP) unpredictability at eastern African Middle Stone Age sites through time, with date ranges of distinct occupations (black lines).

For further exploration of climate predictability, we then analysed the time series produced across the minimum and maximum date range of each archaeological site. This allowed us to assess and plot climate unpredictability in relation to individual occupational date ranges within each site (Figs. 5 and 6). Of the 58 archaeological sites in this dataset (see Fig. 1), 16 from eastern Africa and 15 from northwestern Africa have occupations that, when the date ranges of occupational phases are combined, cover a full precession cycle. Table 1 reports the relative percentages of change versus variability in the time series for each MSA site. Our results from the quantification of variability at the site-level confirms that mean annual temperature unpredictability is increased in eastern Africa compared to northwestern Africa (*p* = 0.129) with between-site variance significantly higher in this region (*p* = 0.022) (Fig. 4). Unpredictability of mean total annual precipitation is however significantly lower in eastern Africa compared to northwestern Africa (*p* = 0.030) and slightly less variable (*p* = 0.501), though only rarely does unpredictability account for more than 10% of the climatic signal in either region (Table 1; Fig. 4). We find no significant differences in the percentages of unpredictability of NPP between (*p* = 0.232) and within (*p* = 0.961) regions when calculated at the site level (Fig. 4), however Figs. 5 and 6 highlight how the overall magnitude of NPP unpredictability within eastern Africa is considerably larger than northwestern Africa, considering that NPP tends to be much higher in this region.

**Table 1:** Percentage of total variance explained by the change versus variability components across the full date range of each middle stone age site in Northwestern (N) and Eastern (E) Africa.

Site	Region	Change bio01	Variability bio01	Change bio12	Variability bio12	Change NPP	Variability NPP
Benzù	N	90.13	9.87	94.14	5.86	90.09	9.91
Bizmoune	N	93.48	4.14	96.74	3.09	91.10	9.37
Contrebandiers	N	87.09	12.91	90.76	9.24	71.26	28.74
Dar es-Soltan 1	N	78.54	21.46	88.68	11.32	92.26	7.74
Dar es-Soltan 2	N	86.07	13.93	90.83	9.17	88.46	11.54
El Harhoura 2	N	70.16	29.84	85.12	14.88	71.58	28.42
El Mnasra	N	78.03	21.97	88.00	12.00	80.17	19.83
Haua Fteah	N	93.85	6.15	99.00	1.00	93.44	6.56
Ifri n’Ammar	N	95.33	4.67	95.44	4.56	91.18	8.82
Jebel Irhoud	N	92.28	7.72	95.64	4.36	89.46	10.54
Rhafas	N	90.05	9.95	92.50	7.50	87.92	12.08
Taforalt	N	87.99	12.01	95.29	4.71	83.19	16.81
Wadi Lazalim - site 15/1	N	83.79	16.21	92.81	7.19	93.01	6.99
Wadi Lazalim - site 16/15	N	94.06	5.94	86.45	13.55	93.88	6.12
Wadi Lazalim - site 16/29	N	96.40	3.60	93.71	6.29	91.42	8.58
Dorothy Garrod Site	E	62.29	37.71	95.77	4.23	87.36	12.64
Eyasi Shore	E	43.84	56.16	95.64	4.36	81.31	18.69
Gademotta	E	82.62	17.38	99.26	0.74	76.73	23.27
Goda Buticha	E	61.62	38.38	94.82	5.18	66.57	33.43
Halibee	E	97.90	2.10	99.24	0.76	93.32	6.68
Kapthurin Formation	E	72.75	27.25	89.98	10.02	90.87	9.13
Karungu	E	53.09	46.91	96.94	3.06	58.02	41.98
Magubike	E	49.63	50.37	73.37	26.63	88.15	11.85
Marmonet Drift	E	93.37	6.63	96.61	3.39	94.29	5.71
Moricho	E	95.52	4.48	94.32	5.68	91.71	8.29
Mumba	E	86.57	13.43	98.47	1.53	81.77	18.23
Ndutu	E	61.81	38.19	95.75	4.25	89.86	10.14
Olorgesailie	E	69.97	30.03	97.03	2.97	71.97	28.03
Omo Kibish	E	95.35	4.65	87.12	12.88	73.36	26.64
Simbi	E	94.09	5.91	95.81	4.19	88.55	11.45
Singa	E	91.92	8.08	96.47	3.53	89.81	10.19

In northwestern Africa, the sites with the most unpredictable temperatures are Atlantic littoral sites El Harhoura 2, El Mnasra and Dar es-Soltan 1, with around 20% of the signal in temperature relating to variability (Table 1; Fig. 5). Interestingly, at coastal El Harhoura 2 cave, a temporal gap in human occupation during Marine Isotope Stage (MIS) 5 is associated with even further increases in the unpredictability in temperature, as well as precipitation (Fig. 5), coinciding with lower sea levels, lower human intensity occupations and increased carnivore inhabitation^8^. Sites with the lowest percentage of temperature variability are Ifri n’Ammar and Wadi Lazalim site 16/29 and site 16/15, at around or less than 5% of the total variance. Conversely, Wadi Lazalim site 16/15 has one of the highest percentages of precipitation variability, with El Harhoura 2, El Mnasra and Dar es-Soltan also having more unpredictable rainfall regimes than other sites in the region (> 10% of the total variance explained by variability) (Table 1; Fig. 5). Haua Fteah and Bizmoune conversely have the most predictable precipitation in northwestern Africa (Table 1; Fig. 5), with the former having a Mediterranean rainfall regime. At Benzù, a chronological hiatus between the two occupations of the site seems to coincide with slight increases in precipitation unpredictability and decrease in temperature unpredictability (Fig. 5). In terms of NPP, Contrebandiers and El Harhoura 2 are the most unpredictable, with around 28% of the total variance relating to variability, whereas Haua Fteah and both sites from Wadi Lazalim site 16/15 and 15/1 are the least unpredictable (Table 1). Overall, the magnitude of unpredictability of NPP in northwestern Africa is considerably lower compared to that at eastern African sites due to plant productivity being more reduced in this region (Figs. 5 and 6).

Within eastern Africa, Eyasi shore and Magubike in Tanzania are the most unpredictable in terms of annual temperature, with > 50% of the time series attributable to the variability component (Table 1; Fig. 6). Halibee, Moricho and Omo Kibish are the most predictable, with < 5% of variance accounted for by variability. Omo Kibish, Magubike and the Kapthurin formation have the highest percentage of variance in precipitation explained by variability (Table 1; Fig. 6). Two distinct phases of human occupation at Omo Kibish during MIS 5 and MIS 6 may be associated with periods of increased precipitation unpredictability (Fig. 6), with nearby woodland along the Omo river potentially providing episodic refugia during more arid downturns^54^. However, almost all of the sites have relatively low amounts of variability in precipitation at less than 10% of the total variance (Table 1). For NPP, Karungu, Goda Butichia and Olorgesaille are the most unpredictable, ranging from ~ 42 − 28% of the total variance, whereas Marmonet Drift, Halibee and Moricho are the most predictable at < 10% (Table 1; Fig. 6). At Moricho, near Kilombe caldera within the central Rift Valley of Kenya, distinct increases in climate unpredictability occur at the same time as a potential gap in human occupation at the site during MIS 6/7 around 180–250 ka (Fig. 6).

## Discussion

We have explored the palaeoenvironmental regimes experienced during the MSA between two different biogeographic regions of Africa: northwestern and eastern Africa. Our results, summarised in Table 2, demonstrate key differences in the nature and tempo of climatic change between these two regions that likely had major implications for technological investment, innovation and diversity in MSA-making populations^33^. We recognise that the site distributions analysed here may be influenced by taphonomic factors and/or archaeological practice; nonetheless, our results highlight some clear regional differences in environmental conditions occupied, which are shown to be largely robust in sensitivity analyses. Below we discuss how our results might help to understand the impact of climatic fluctuation on behavioural diversification in early *Homo sapiens*, while we emphasise that this is still largely a theoretical exercise until rigorous African continental-scale quantitative comparisons of artefacts and assemblages are possible.

**Table 2:** Summary of the results, with statistically significant results marked with an asterisk (*).

Region	Test	Temperature			Precipitation			NPP	
		Annual	Seasonality	Predictability	Annual	Seasonality	Predictability	Annual	Predictability
Eastern Africa	Mean compared to northwestern occupations	Warmer *	Less seasonal *	Less predictable	Wetter *	Less seasonal	More predictable	More productive *	Less predictable
	Regional variability compared to northwestern occupations	More variable *	Less variable	More variable*	More variable *	Less variable *	Less variable	More variable *	Less variable
	Compared to background in eastern Africa	Colder *	Less seasonal *	NA	Wetter *	Less seasonal *	NA	More productive *	NA
North Western Africa	Mean compared to eastern occupations	Colder *	More seasonal *	More predictable	Drier *	More seasonal	Less predictable	Less productive *	More predictable
	Regional variability compared to eastern occupations	Less variable *	More variable	Less variable*	Less variable *	More variable *	More variable	Less variable *	More variable
	Compared to background in northwestern Africa	Colder *	Less seasonal *	NA	Wetter *	Less seasonal *	NA	More productive *	NA

In modern hunter-gatherers, temperature seasonality and predictability are increasingly influential over technological complexity (defined as tools with a higher number of individual components, made for specific tasks^21^) as one moves further away from the equator^22^. Determining tool modularity from the archaeological records is challenging due to incomplete preservation and the need for different quantification systems for measuring complexity in lithic reduction sequences^55,56^. Yet, in hunter-gatherers, more complex tools and toolkits tend to be those that are highly curated to produce the greatest returns for specific activities when the cost of resource acquisition failure is high^21,22^, providing an interpretative link relevant to expressions of specialisation in the MSA archaeological record. Our results show that, in northwestern Africa, MSA occupations are associated with higher temperature seasonality and predictability compared to eastern Africa, which instead shows generally much better conditions for plant availability across the year and is extremely ecologically variable. Based on observations from the ethnographic record, one can thus make several hypotheses about the patterning of archaeological variability in these areas during the MSA and the purported links between technology and ecological risk.

For example, one could suggest that sparse populations occupying northwestern Africa at various times during the Middle to Late Pleistocene may have converged on specific (likely hunting-based^8,57,58^) strategies to ensure resource capture across seasonal landscapes. This is supported by functional analyses and faunal evidence from lithic assemblages labelled as ‘Aterian’, which suggest that tanging modifications may have been crucial technological advances for highly mobile groups who followed animal herds^59^. A strong reliance on hunting is also supported by a distinct increase in the utilisation of animal materials within the Aterian technical system at Ifri n’Ammar^60^. While typo-technological consistency of coastal Aterian assemblages from El Harhoura 2 and El Mnasra has been noted within the same chronological window (MIS 5/4), tanged tools are absent at the former and present at the latter^61,62^. Various hypotheses have been presented, including differences in occupation intensity and site function^57,63^. For example, human occupation seems more intense in the MIS 5 units than carnivores at these sites, with increased exploitation of coastal resources (such as molluscs), probably explained by the proximity to the seashore during periods of higher sea levels^8^. Coastal occupations likely required other technological adaptations (such as tools to extract limpet shells from rocks^64^), perhaps reducing the emphasis on risk mitigation strategies associated with hunting in some instances. Additionally, occupations in the coastal Rabat-Témera region (i.e. El Harhoura 1 and 2, El Mnasra, Contrebandiers, Dar es Soltane 1 and 2) are associated with relatively high temperature unpredictability compared to other sites in northwestern Africa; conditions that may have hindered the accumulation of landscape knowledge across multiple generations. However, modelling work shows that this area likely fell within a pocket of precipitation refugia along the Atlantic and Mediterranean coasts^39,42^where enough rainfall for human populations to persist was maintained. Technological know-how may have been shared via population networks organised around refugial zones, shared environments and perennial water bodies, the latter of which would have also served to attract fauna^65,66^. Extensive mobility between coastal and inland areas is supported by the presence of marine shells at Ifri n’Ammar, > 280 km from the Atlantic and > 40 km from the Mediterranean^67^. The maintenance of certain risk-management strategies was therefore likely specific to certain ecological challenges, such as periods of reduced faunal availability or changes in sea level. Another potential example of such risk mitigation behaviours during the MSA could be ‘Nubian’ reduction methods found in arid regions across Africa (including eastern Africa) and Southwest Asia, which have been hypothesised to be both technologically more efficient and produce pointed tools with clear functional advantages over other reduction systems^68^.

During the Middle to Late Pleistocene, environments in eastern Africa were highly varied across space and time^26^. At Olorgesailie in the southern Kenyan rift, increased fluctuations in resource variability coincide with the transition from the Acheulean to the MSA ca. 400 ka^15,16^. High spatiotemporal variability, as well as areas of extremely unpredictable temperatures and NPP, may therefore help explain the emergence of intra-regional variability in material culture that seems to characterise the MSA of the region. A correlation between relatively ‘simple’ generalist technologies and high temperature variability at sites in southern Tanzania is supported by lithic assemblages from Magubike, which show a lack of retouch and reliance on generic tools used for multiple function. Werner and Willoughby^69^ interpreted this as a reflection of the increasingly unpredictable resources in the area. Similarly, assemblages from Eyasi Shore are described as ‘undiagnostic’ in nature^70^. However, temperature may be less important in terms of ecological risk in most places considering that precipitation predictability is largely favourable, as this has more of an impact on plant distributions and food availability in inter-tropical climates^71^. This is highlighted by the fact that both lithic assemblage variation^26,27^and the diversity of pointed tool forms^72^appear to be responsive to spatiotemporal differences in precipitation rather than temperature in tropical Africa. For example, points from broadly similar assemblages at Omo Kibish and Gademotta in Ethiopia have clear frequential and technological differences^54^, with our results suggesting the former is associated with considerably lower and more unpredictable rainfall. Our results also support that MSA populations in eastern Africa occupied largely productive climatic conditions that would have supported dense and diverse tropical shrubland plants that changed through time. Such settings may consequently have fostered plasticity in the deployment of technological behaviour from the MSA repertoire^48,73^, leading to diversity among nearby groups as they dealt with changing resource bases primarily mediated by local patterns of rainfall^26^. Areas with more plant productivity may also have facilitated greater and more stable population densities. This may have provided demographic conditions amenable to the early emergence of innovations associated with the Later Stone Age in this region^74^, some of which (e.g. blades) may relate to new behavioural strategies for dealing with periodic increases in seasonality^75^.

Overall, motivations for investing in specific types, styles or levels of complexity in toolmaking were likely highly variable in response to specific environmental challenges and mediated by social practice at different spatial and temporal scales. This is because technological innovations should only be developed and maintained when the expected returns from the required investment exceed the potential cost associated with the risk of not having done so^7,33^.We have explored here how environmental productivity, seasonality, and predictability may have impacted technological diversification during the MSA, utilising the theoretical framework presented by Clark and Linares-Matás^33^. While our results are compelling in terms of their complementarity with ethnographic datasets^22^, our hypotheses need to be tested explicitly using comparative archaeological data and robust quantitative approaches. It is also important to note that Clark and Linares-Matás^33^focus their framework on climatic parameters that affect a single generation, such as intra- and inter-annual seasonality and predictability, whereas we report climate conditions within and across millennia based on the chronological resolution of the model employed^50^. Indeed, the ethnographic record emphasises the importance of seasonality for understanding modern human behavioural adaptation to diverse environments, however it is difficult to identify and study in the archaeological record. At the same time, we should expect it to play an important role in structuring the archaeological record^33^and a number of authors are now successfully working on ways of studying seasonality in deep time across Africa on both short and long timescales (e.g^75–77^). Moreover, increased unpredictability in resource distributions between years constrains the amount of landscape knowledge that can be accumulated; however, knowledge can also be built up over multiple years and generations via cumulative culture^55^. Indeed, the MSA record seems to represent the flexible expression of particular subsets of this behavioural repertoire^73^. Across more extended evolutionary timescales, inconsistency in selective pressures therefore favours structures and behaviours responsive to complex environmental diversity, consequently leading to ‘generalism’ rather than ‘specialism’ tendencies^14^. Future research could seek to quantify climate seasonality and predictability at a finer chronological scale to capture instability experienced at the population-level, though current limitations of the palaenvironmental and archaeological records makes this a challenging endeavour at this spatiotemporal scale.

## Conclusion

Intrinsic properties of the landscape, such as its resource abundance and diversity, as well as within- and across-millennia variability, show distinct differences between eastern and northern areas of Africa during the Middle to Late Pleistocene. Compared to those in eastern Africa, bioclimatic models suggest that northwestern MSA occupations are generally associated with colder, drier and less productive environments, albeit wetter (and still cooler) than background environmental settings, with more seasonal temperatures but generally predictable climates across millennia.

Based on observations from modern hunter-gatherers^22^, temperature seasonality at more extreme latitudes (particularly in areas with lower plant availability) had the potential to impose stronger selective pressures on technological variability due to shorter periods of game availability^23^. Investment in certain types of MSA tools or toolkits at particular times and in particular places, such as ‘Aterian’ tanged tools or ‘Nubian’ reduction methods, may have been stimulated in response to distinct sources of ecological risk, leading to technological specialisation with distinct signatures in the archaeological record that persist over long periods of time. In tropical equatorial regions, precipitation and its spatiotemporal variability are likely to act as a stronger mediator of adaptive responses due to their impact on plant distributions^71^. Unpredictable temperatures and NPP in combination with spatial topographic diversity may have played some role in the emergence and divergence of MSA toolkits within eastern Africa, by favouring the development of diverse, generalised technological strategies that can be applied across a variety of foraging settings^14–16^.

Considering our results and interpretations, we stress that ecological risk was likely not experienced in the same way nor extent across the large and diverse African continent, as selective pressures for behavioural adaptation act at different scales, on different technological elements, and in different biogeographic and ecological contexts. Variable sources of ecological risk between and within regions were therefore potentially major drivers of cultural diversification between MSA-making populations. This has vital implications for theoretical models of pan-African human evolution, particularly for understanding how regional groups and their interconnectivity were structured in relation to changing ecological conditions^9^.

## Methods

### Datasets

We aggregated published datasets of MSA site coordinates (*N* = 58; Fig. 1) and dates of human occupation from northwestern (*n* = 111) and eastern (*n*= 112) Africa^48,49^. Both datasets were selected with the same criteria to ensure comparability. For northwestern Africa, we subsetted ‘contextual’ dates (i.e. those directly associated with MSA human activity) from the inventory of dates presented by Boisard and Ben-Arous^48^. For eastern Africa, we included all occupations associated with MSA assemblages reported by Blinkhorn and Grove^49^ and an additional 28 occupations that either (1) are newly published, (2) have been chronologically revised and/or (3) are occupations that haven’t previously merited inclusion in analyses based on the low availability of lithic data (which is not focussed on specifically in this inter-region palaeoenvironmental comparison, as there currently is no comparable dataset for northwestern Africa). Our final dataset comprised 223 dated occupations from across both regions, which we subsequently reduced to 165 unique occupations for our statistical analyses (eastern Africa = 59, northwestern Africa = 106), as many distinct occupations have the same potential date range and location, particularly in eastern Africa.

To estimate the date ranges of each archaeological layer, we followed established protocols^26,39,42,48,72,73^. We used the standard deviation of each date (minimum and maximum date), and for multi-dated archaeological layers, the maximum age estimate was determined based on the oldest date and the minimum age estimate based on the youngest date. We also determined the mid-point of this date range (what we refer to as the ‘mid-age’), which we use in statistical analysis to compare between and within regions. In northwestern Africa, sites selected are dated from ca. 332 − 25 ka based on the mid-age, with those from eastern Africa similarly dated from ca. 308 − 25 ka.

As shown in Fig. 1, there are important distributional differences in the datasets that may contribute to some of the patterns observed. In northwestern Africa, there are fewer sites (*N* = 21) with multiple occupations within a tighter latitudinal band. Comparatively, in eastern Africa, there are more sites (*N* = 37) with fewer repeat occupations and a wider latitudinal range, crossing the equator. The fact that the eastern African database contains sites on either side of the equator has important ramifications for the analyses. Axial precession, a particularly important influence on tropical climate, increases seasonal contrasts in one hemisphere while simultaneously decreasing them in the other. Precession mediates precipitation in a similar way, for example via shifting monsoon intensities; in Fig. 2, the eastern African sites show clear precessional (~ 23,000 years) periodicity in precipitation, but with sites in the northern hemisphere showing a pattern approximately the inverse of that shown by southern hemisphere sites. Both regions have occupations distributed across the chronological range of the dataset, increasing in density from MIS 5 onwards (Figs. 2 and 3).

### Climate parameters

Using the site coordinates and the date range of each occupation, we extracted mean annual temperature (bio01), temperature seasonality (bio04; standard deviation of monthly temperature averages, multiplied by 100), mean total annual precipitation (bio12), precipitation seasonality (bio15; coefficient of variation of monthly precipitation totals, expressed as a percentage) and mean net primary productivity (NPP) from a high-resolution statistics-based reconstructed climatic time series based on the HadCM3 global circulation model^52^ using the *pastclim*R package^78^. We selected 1000-year time slices from this model time series from across the date range of each occupation, and used these to calculate a minimum, maximum and mid-age of each variable for each occupation. We recognise that these date ranges represent dating error as opposed to the potential chronological span of human activity at the sites, however we use these as temporal boundaries within which to explore climatic change through time in specific locations associated with human activity.

We applied the model at its original resolution of 30-arcminutes, though set the ‘buffer’ parameter to ‘directions = 8’ to account for potential landscape variability in climate across neighbouring cells^79^. Coordinates for two sites, Benzú (North Africa) and Rusinga (East Africa) were moved to the nearest cell on land to avoid issues with sea-level/water body masking in the original model output.

### Comparative analysis

To investigate differences in climates between eastern and northwestern Africa, we used mid-age estimates of the five climatic parameters as well as their variability across the full date range of each occupation. To plot climatic change in relation to MIS, we utilised the Lisiecki and Raymo^80^ dataset extracted from the *gsloid*R package^81^. As our data are not normally distributed, we employed non-parametric Mann-Whitney U Test and Ansari-Bradley tests to test for differences in median and variance between the two regions, with a p-value < 0.05 deemed to be statistically significant. We also calculated the coefficient of variation, which is the ratio of the standard deviation to the mean, expressed here as a percentage; this is a standardised measure of intra-region variability which is not sensitive to the distribution of the data.

To assess the robusticity of our statistical tests that compare climatic values from the two regions using the mid-age, we performed sensitivity analyses; this involved running 1000 iterations of each statistical test, randomly permuting the time slice from which the climatic values are extracted across the date range (i.e., every 1000-year time slice between the minimum age and maximum date). From the results of each permutation, we recorded the coefficient and p-value of the test. We then examined the distribution of the results in relation to those produced using the mid-age; if the majority of iterations produced comparable results to that produced by the mid-age, we deemed our conclusions to be robust.

We also explored climatic conditions at MSA occupations in relation to the regional background. To do this, we extracted the climatic values at the MSA occupations in the dataset at the mid-age, and then compared them to random samples of values extracted from across each region across the MSA (temporally defined as the minimum date to the maximum date of the whole dataset). Following previous work^26,42^, we defined eastern Africa as (30, 55, −9, 20) and northwestern Africa as (−15, 35, 18, 39), cropped using a shape file of the African continent using the *rnaturalearth*R package^82^. We performed 1000 permutations of the 59 dated occupations in eastern Africa and 106 in northwestern Africa, randomly sampling the same number of cells as occupations through space and time. We then tested whether there are significant differences between the random samples and that produced by the mid-age values at actual MSA occupations.

### Quantifying climate predictability

To calculate inter-millennial predictability, we utilised the change/variability decomposition (CVD) algorithm for differentiating between change and variability components in climatic time series^53^. This algorithm uses singular spectrum analysis to decompose the time series into a series of empirical orthogonal functions (EOFs). The EOFs are then recombined one by one, in descending order of their associated eigenvalues. This produces two sets, one consisting of EOFs 1 to $\:w$ and the other consisting of EOFs $\:w+1$ to $\:M$; each time a new EOF is added to the first set, the value of w increases by 1. The CVD determines the smallest value of w for which the set of EOFs $\:w+1$ to $\:M$ is consistent with white noise. The white noise test is conducted in the frequency domain by employing the 95% confidence interval around the theoretical expectation for the power spectrum of a white noise series, using a discrete Fourier transform of the summed $\:w+1$ to $\:M$ EOFs.

**Fig. 7:**
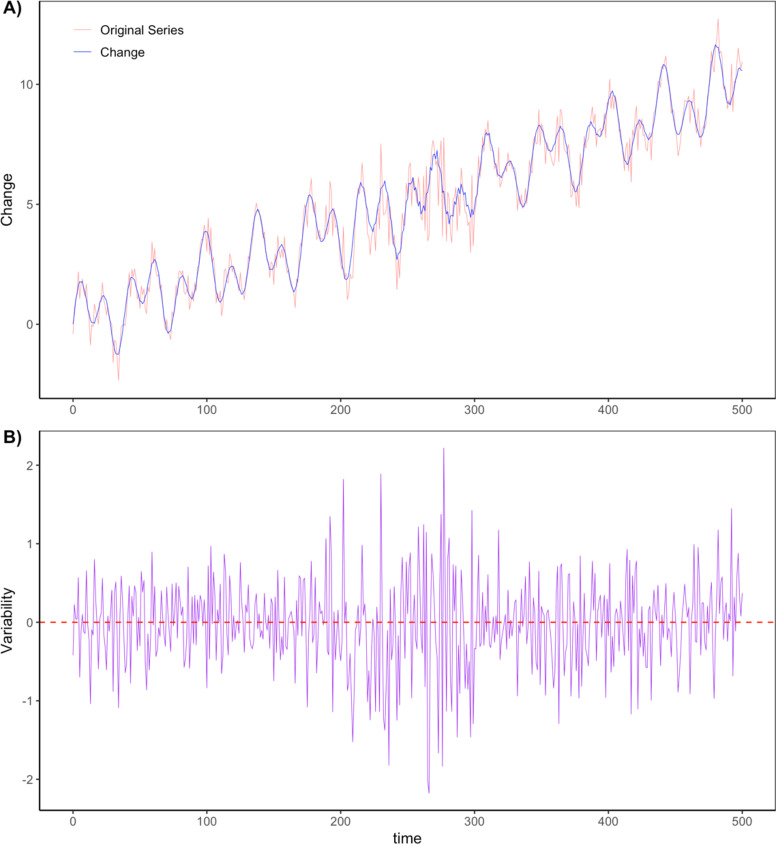
An example of the change/variability decomposition algorithm^53^ for a randomly generated time series with (A) the change component (blue) superimposed onto the original time series (red), and (B) the change-corrected variability component (purple).

Following this procedure, the first set of EOFs is summed to represent the ‘change’ component and the second set is summed to represent the ‘variability’ component (Fig. 7). Adding these two components back together reproduces the original time series. With the full set of eigenvalues normalised to sum to 100, the sums of the normalised eigenvalues associated with the first and second sets of EOFs give the percentages of variance accounted for by the change and variability components respectively. These metrics are directly comparable between time series because they are calculated on the de-trended, z-scored versions of the time series used for the singular spectrum analysis, though they do not take into account any variance introduced to the time series by long-term trends occurring over periods greater than the embedding dimension ($\:M$).

The CVD algorithm requires the user to choose an embedding dimension; Grove^53^ suggests this should be selected based on a trade-off between the need to capture sufficient information about low-frequency components (favouring large $\:M$) and the need for sufficient repetitions of the embedding window over the total length of the time series (favouring small $\:M$). If capturing a particular frequency is important to the analysis, $\:M$ should be set to be at least as large as the reciprocal of that frequency. Given the importance the precession cycle to tropical African climate, we calculated the change and variability components of mean annual temperature (bio01), mean annual precipitation (bio12) and net primary productivity (npp) using $\:M$ = 23 = 23,000 years.

Defining variability as per the CVD algorithm is particularly relevant to the current analyses, as the lack of autocorrelation in a white noise time series corresponds directly to the unpredictability we aim to measure. We therefore use the percentage of variance accounted for by the ‘variability’ component of the CVD output as our proxy for predictability, with locations where this percentage is higher deemed as having more unpredictable climates. Finally, we compared the average percentage of variance explained by the variability component between regions.

## Electronic supplementary material

Below is the link to the electronic supplementary material.

Supplementary Material 1

## Data Availability

All code and data to perform the analyses can be found at https://osf.io/cx9uk/?view_only=b30d71bd76be4e22aaf0ee940fb5c166, and were made accessible for the review of this manuscript.

## References

[1] Richter, D. et al. The age of the hominin fossils from Jebel Irhoud, Morocco, and the origins of the middle stone age. *Nature* **546**, 293–296. 10.1038/nature22335 (2017). 28593967 10.1038/nature22335

[2] Hublin, J. J. et al. New fossils from Jebel Irhoud, Morocco and the pan-African origin of homo sapiens. *Nature* **546**, 289–292. 10.1038/nature22336 (2017). 28593953 10.1038/nature22336

[3] Brooks, A. S. et al. Long-distance stone transport and pigment use in the earliest middle stone age. *Science* **360**, 90–94. 10.1126/science.aao2646 (2018). 29545508 10.1126/science.aao2646

[4] McBrearty, S. & Brooks, A. S. The revolution that wasn’t: a new interpretation of the origin of modern human behavior. *J. Hum. Evol.* **39**, 453–563. 10.1006/jhev.2000.0435 (2000). 11102266 10.1006/jhev.2000.0435

[5] Clark, J. D. The middle stone age of East Africa and the beginnings of regional identity. *J. World Prehist*. **2**, 235–305 (1988).

[6] Scerri, E. M. L. & Will, M. The revolution that still isn’t: the origins of behavioural complexity in homo sapiens. *J. Hum. Evol.* **179**, 103358. 10.1016/j.jhevol.2023.103358 (2023). 37058868 10.1016/j.jhevol.2023.103358

[7] Timbrell, L. Ecology and demography of early homo sapiens: a synthesis of archaeological and climate data from Eastern Africa. *Azania* **59** (1), 76–110. 10.1080/0067270X.2024.2307790 (2024).

[8] Campmas, E. Integrating Human-Animal relationships into new data on Aterian complexity: a paradigm shift for the North African middle stone age. *Afr. Archaeol. Rev.* **34**, 469–491. 10.1007/s10437-017-9273-z (2017).

[9] Scerri, E. M. L. et al. Did our species evolve in subdivided populations across Africa, and why does it matter? *Trends Ecol. Evol.* **33**, 582–594. 10.1016/j.tree.2018.05.005 (2018). 30007846 10.1016/j.tree.2018.05.005 PMC6092560

[10] Ben Arous, E., Falguères, C., Nespoulet, R. & El Hajraoui, M. Review of chronological data from the Rabat-Témara caves (Morocco): implications for understanding human occupation in Northwestern Africa during the Late Pleistocene. In A. Leplongeon, M. Goder-Goldberger, D. Pleurdeau (Eds) *Not Just a Corridor. Human occupations of the Nile Valley and neighbouring regions between 75,000 and 15,000 years ago*. Natures en Sociétés, pp. 177–201. (2020).

[11] Bouzouggar, A. et al. 82,000-year-old shell beads from North Africa and implications for the origins of modern human behavior. *Proc. Natl. Acad. Sci. USA*. **104** (24), 9964–9969. 10.1073/pnas.0703877104 (2007). 17548808 10.1073/pnas.0703877104 PMC1891266

[12] Texier, P. J. et al. From the cover: A Howiesons Poort tradition of engraving ostrich eggshell containers dated to 60,000 years ago at Diepkloof rock shelter, South Africa. *Proc. Natl. Acad. Sci. USA*. **107** (14), 6180–6185. 10.1073/pnas.0913047107 (2010). 20194764 10.1073/pnas.0913047107 PMC2851956

[13] Shea, J. J. A. & Generic, M. S. A. What problems will it solve, and what problems will it create? *Azania* **59** (1), 160–172. 10.1080/0067270X.2024.2306078 (2024).

[14] Potts, R. Variability selection in hominid evolution. *Evol. Anthropol.* **7** (3), 81–96. 10.1002/(SICI)1520-6505(1998)7:3%3C81::AID-EVAN3%3E3.0.CO;2-A (1998).

[15] Potts, R. et al. Environmental dynamics during the onset of the middle stone age in Eastern Africa. *Science* **360**, 86–90. 10.1126/science.aao2200 (2018). 29545506 10.1126/science.aao2200

[16] Potts, R. et al. Increased ecological resource variability during a critical transition in hominin evolution. *Sci. Adv.* **6**, eabc8975. 10.1126/sciadv.abc8975 (2020). 33087353 10.1126/sciadv.abc8975 PMC7577727

[17] Klein, R. G. Southern Africa and modern human origins. *J. Anthropol. Res.* **57**, 1–16 (2001).

[18] Foley, R. A. & Mirazón Lahr, M. Variable cognition in the evolution of Homo: biology and behaviour in the African Middle Stone Age. In J Cole, J McNabb & M Grove (Eds) *Landscapes of Human Evolution. Contributions in honour of John Gowlett*, pp. 124–140. Archaeopress. (2020).

[19] Wilkins, J. & Schoville, B. J. Did climate change make *Homo sapiens* innovative, and if yes, how? Debated perspectives on the African pleistocene record. *Quat Sci. Adv.* **14**, 100179. 10.1016/j.qsa.2024.100179 (2024).

[20] Lombard, M. & Parsons, I. What happened to the human Mind after the Howiesons poort?? *Antiquity* **85**, 1433–1443. 10.1017/S0003598X00062153 (2011).

[21] Oswalt, W. H. *An anthropological analysis of food-getting technology* (Wiley-Interscience, 1976).

[22] Clark, J., Timbrell, L., Paris, S. & Linares-Matás, G. Complex landscapes of cultural evolution: re exploring the socioecological drivers of technological variation in modern hunter-gatherers. *Evolutionary Hum. Sci.* (under review).

[23] Torrence, R. Hunter-gatherer technology: macro-and microscale approaches. In *Hunter-Gatherers: an Interdisciplinary Perspective* (eds Panter-Brick, C. et al.) 73–98 (Cambridge University Press, 2001).

[24] Collard, M., Buchanan, B., Morin, J. & Costopoulos, A. What drives the evolution of hunter–gatherer subsistence technology? A reanalysis of the risk hypothesis with data from the Pacific Northwest. *Philos. Trans. R Soc. Lond. B Biol. Sci.* **366**, 1129–1138 (2011). 10.1098%2Frstb.2010.0366. 21357235 10.1098/rstb.2010.0366 PMC3049102

[25] Thompson, J. C. et al. Ecological risk, demography and technological complexity in the late pleistocene of Northern Malawi: implications for geographical patterning in the middle stone age. *J. Quat Sci.* **33**, 261–284. 10.1002/jqs.3002 (2018).

[26] Timbrell, L., Grove, M., Manica, A., Rucina, S. & Blinkorn, J. A Spatiotemporally explicit paleoenvironmental framework for the middle stone age of Eastern Africa. *Sci. Rep.* **12**, 3689. 10.1038/s41598-022-07742-y (2022). 35256702 10.1038/s41598-022-07742-y PMC8901736

[27] Padilla-Iglesias, C., Grove, M. & Blinkhorn, J. Ecological drivers of hunter-gatherer lithic technology from the middle and later stone age in central Africa. *Quat Sci. Rev.* **322**, 108390 (2023).

[28] Powell, A., Shennan, S. & Thomas, M. G. Late pleistocene demography and the appearance of modern human behavior. *Science* **324**, 1298–1301. 10.1126/science.1170165 (2009). 19498164 10.1126/science.1170165

[29] Grove, M. Population density, mobility, and cultural transmission. *J. Archaeol. Sci.* **74**, 75–84. 10.1016/j.jas.2016.09.002 (2016).

[30] Grove, M. Hunter-gatherers adjust mobility to maintain contact under Climatic variation. *J. Archaeol. Sci. Rep.* **19**, 588–595. 10.1016/j.jasrep.2018.04.003 (2018).

[31] Henrich, J. et al. Understanding cumulative cultural evolution. *Proc. Natl. Acad. Sci. USA*. **113** (44), E6724–E6725. 10.1073/pnas.1610005113 (2016). 27791123 10.1073/pnas.1610005113 PMC5098628

[32] Clark, J. & Linares-Matás, G. J. Seasonality and lithic investment in the Oldowan. *J. Paleolit Archaeol.* **6**10.1007/s41982-023-00165-9 (2023).

[33] Clark, J. & Linares-Matás, G. J. When to generalise and when to specialise? Climate change and hominin biocultural adaptability in the African early and middle stone age. *Quat Sci. Adv.* **15**, 100218. 10.1016/j.qsa.2024.100218 (2024).

[34] Burke, A. et al. The archaeology of climate change: a blueprint for integrating environmental and cultural systems. *Nat. Commun.* (under review). 10.1038/s41467-025-60450-9 PMC12166059 40514402

[35] Moran, N. A. The evolutionary maintenance of alternative phenotypes. *Am. Nat.* **139**, 971–989. 10.1086/285369 (1992).

[36] Gienapp, P., Teplitsky, C., Alho, J. S., Mills, J. A. & Merilä, J. Climate change and evolution: disentangling environmental and genetic responses. *Mol. Ecol.* **17**, 167–178. 10.1111/j.1365-294X.2007.03413.x (2008). 18173499 10.1111/j.1365-294X.2007.03413.x

[37] Chevin, L. M., Lande, R. & Mace, G. M. Adaptation, plasticity, and extinction in a changing environment: towards a predictive theory. *PLoS Biol.* **8**, e1000357. 10.1371/journal.pbio.1000357 (2010). 20463950 10.1371/journal.pbio.1000357 PMC2864732

[38] Blome, M. W., Cohen, A. S., Tryon, C. A., Brooks, A. S. & Russell, J. The environmental context for the origins of modern human diversity: a synthesis of regional variability in African climate 150,000–30,000 years ago. *J. Hum. Evol.* **62**, 563–592. 10.1016/j.jhevol.2012.01.011 (2012). 22513381 10.1016/j.jhevol.2012.01.011

[39] Blinkhorn, J., Timbrell, L., Grove, M. & Scerri, E. Evaluating refugia in recent human evolution in Africa. *Philos. Trans. R Soc. Lond. B Biol. Sci.* **377**, 1849. 10.1098/rstb.2020.0485 (2022). 10.1098/rstb.2020.0485 PMC8899617 35249393

[40] Ossendorf, G. et al. Middle stone age foragers resided in high elevations of the glaciated Bale mountains, Ethiopia. *Science* **365**, 583–587. 10.1126/science.aaw8942 (2019). 31395781 10.1126/science.aaw8942

[41] Schaebitz, F. et al. Hydroclimate changes in Eastern Africa over the past 200,000 years May have influenced early human dispersal. *Commun. Earth Environ.* **2**10.1038/s43247-021-00195-7 (2021).

[42] Boisard, S., Wren, C., Timbrell, L. & Burke, A. Climate frameworks for the middle stone age and later stone age in Northwest Africa. *Quat Int.* **716**, 109593 (2025).

[43] Marquer, L. et al. The first use of olives in Africa around 100,000 years ago. *Nat. Plants*. **8**, 204–208. 10.1038/s41477-022-01109-x (2022). 35318448 10.1038/s41477-022-01109-x

[44] Stoetzelet al. et al. Late cenozoic micromammal biochronology of Northwestern Africa. *Palaeogeogr Palaeoclimatol Palaeoecol*. **392**, 359–381. 10.1016/j.palaeo.2013.09.026 (2013).

[45] Lalis, A. et al. Out of Africa: demographic and colonization history of the Algerian mouse (*Mus spretus* Lataste). *Heredity* **122**, 150–171. 10.1038/s41437-018-0089-7 (2019). 29795180 10.1038/s41437-018-0089-7 PMC6327062

[46] Kaboth-Bahr, S. et al. Paleo-ENSO influence on African environments and early modern humans. *Proc. Natl. Acad. Sci. USA*. **118** (e2018277118). 10.1073/pnas.2018277118 (2021). 10.1073/pnas.2018277118 PMC8201937 34074756

[47] 47. Ben Arous, E., Boisard, S. & Leplongeon, A. The Upper Pleistocene Archaeology of northern Africa (Middle and Later Stone Age, from the western Maghreb to the Nile Valley). In: Elias, S. (eds.) *Encyclopedia of Quaternary Science, 3rd Edition*. vol. 1, pp. 108–122. UK: Elsevier. dx.doi.org/10.1016/B978-0-323-99931-1.00241-5 (2025).

[48] Blinkhorn, J. & Grove, M. Explanations of variability in middle stone age stone tool assemblage composition and Raw material use in Eastern Africa. *Archaeol. Anthropol. Sci.* **13**, 14. 10.1007/s12520-020-01250-8 (2021).

[49] Boisard, S., Ben Arous, E. A. & Critical Inventory Associated chronology of the middle stone age and later stone age in Northwest Africa. *J. Open. Archaeol. Data*. **12**10.5334/joad.121 (2024).

[50] Scerri, E. M. L. & Spinapolice, E. E. Lithics of the North African middle stone age: assumptions, evidence and future directions. *J. Anthropol. Sci.* **97**, 9–43. 10.4436/jass.97002 (2017). 10.4436/JASS.97002 31294697

[51] Shea, J. J. *Prehistoric Stone Tools of Eastern Africa: A Guide* (Cambridge University Press, 2020).

[52] Krapp, M., Beyer, R. M., Edumundson, S. L., Valdes, P. J. & Manica, A. A statistics-based reconstruction of high-resolution global terrestrial climate for the last 800,000 years. *Sci. Data*. **8**, 228. 10.1038/s41597-021-01009-3 (2021). 34453060 10.1038/s41597-021-01009-3 PMC8397735

[53] Grove, M. Climatic change and Climatic variability: an objective decomposition. *Quat Sci. Rev.* **271**10.1016/j.quascirev.2021.107196 (2021).

[54] Shea, J. J. The middle stone age archaeology of the lower Omo Valley Kibish formation: excavations, lithic assemblages, and inferred patterns of early homo sapiens behavior. *J. Hum. Evol.* **55** (3), 448–485. 10.1016/j.jhevol.2008.05.014 (2008). 18691735 10.1016/j.jhevol.2008.05.014

[55] Paige, J. & Perreault, C. 3.3 Million years of stone tool complexity suggests that cumulative culture began. *Proc. Natl. Acad. Sci. USA*. **121** (26), e2319175121. 10.1073/pnas.2319175121 (2024). 38885385 10.1073/pnas.2319175121 PMC11214059

[56] Perreault, C., Brantingham, P. J., Kuhn, S. L., Wurz, S. & Gao, X. Measuring the complexity of lithic technology. *Curr. Anthropol.* **54** (S8), S397–S406. 10.1086/673264 (2013).

[57] Nespoulet, R. et al. Palaeolithic and neolithic occupations in the Temara region (Rabat, Morocco): recent data on hominin contexts and behavior. *Afr. Archaeol. Rev.* **25** (1–2), 21–39. 10.1007/s10437-008-9025-1 (2008).

[58] Campmas, E. et al. Comportements de subsistance à l’Atérien et au Néolithique au Maroc Atlantique: premiers résultats de l’étude taphonomique et archéozoologique des faunes d’El Harhoura 2 (Région de Témara, Maroc). Actes du colloque RQM4, Le Quaternaire marocain dans son contexte méditerranéen, Oujda, 15–17 Novembre. pp. 236–254 (2008).

[59] Tomasso, S. & Rots, V. What is the use of shaping a Tang?? Tool use and hafting of Tang?ed tools in the Aterian of Northern Africa. *Archaeol. Anthropol. Sci.* **10**, 1389–1417. 10.1007/s12520-016-0448-3 (2018).

[60] Tomasso, S. *What is new in the Aterian? A functional view on tool use, hafted stone tool technologies and assemblage variability at Ifri n’Ammar within the context of the Northwest African Middle Stone Age* (Forschungen zur Archäologie Außereuropäischer Kulturen, 2024).

[61] Stoetzel, E. et al. Context of modern human occupations in North Africa: contribution of the Témara caves data. Northwest African prehistory: recent work, new results and interpretations. *Quat Int.* **320**, 143–161 (2014).

[62] Campmas, E. et al. Were upper pleistocene human/non-human predator occupations at the Témara caves (El Harhoura 2 and El Mnasra, Morocco) influenced by climate change? *J. Hum. Evol.* **78**, 122 (2015). 25281232 10.1016/j.jhevol.2014.08.008

[63] Dibble, H. et al. New excavations at the site of contrebandiers cave, Morocco. *Palaeoanthropology* **2012**, 145–201 (2012).

[64] Nouet, J. et al. Limpet shells from the Aterian level 8 of El Harhoura 2 cave (Témara, Morocco): preservation state of Crossed- foliated layers. *PLoS ONE*. **10** (9), e0137162 (2015). 26376294 10.1371/journal.pone.0137162 PMC4574309

[65] Scerri, E. M. L., Drake, N. A., Jennings, R. & Groucutt, H. S. Earliest evidence for the structure of homo sapiens populations in Africa. *Quat Sci. Rev.* **101**, 207–216. 10.1016/j.quascirev.2014.07.019 (2014).

[66] Drake, N. A., Blench, R. M., Armitage, S. J., Bristow, C. S. & White, K. H. Ancient watercourses and biogeography of the Sahara explain the peopling of the desert. *Proc. Natl. Acad. Sci. USA.* **108**, 358e462. 10.1073/pnas.1012231108 (2011). 10.1073/pnas.1012231108 PMC3021035 21187416

[67] Nami, M. & Moser, J. *La Grotte D’Ifri N’Ammar. Le Paléolithique Moyen. Forschungen Zur Archäologie Außereuropäischer Kulturen* Vol. 9 (Reichert, 2010).

[68] Samawi, O. & Hallinan, E. More than surface finds: Nubian Levallois core metric variability and site distribution across Africa and Southwest Asia. *J. Paleo Arch.* **7**10.1007/s41982-024-00192-0 (2024).

[69] Werner, J. J. & Willoughby, P. R. Middle stone age technology and cultural evolution at Magubike rockshelter, Southern Tanzania. *Afri Archaeol. Rev.* **34**, 249–273 (2017).

[70] Dominguez-Rodrigo et al. The archaeology of the middle pleistocene deposits of lake Eyasi. *Tanzan. J. Afr. Archaeol.* **5** (1), 47–78 (2007).

[71] Boyle, A. W., Shogren, E. S. & Brawn, J. D. Hygric niches for tropical endotherms. *Trends Ecol. Evol.* **35**, 10. 10.1016/j.tree.2020.06.011 (2020). 32693967 10.1016/j.tree.2020.06.011

[72] Timbrell, L. et al. Stone point variability reveals Spatial, chronological, and environmental structuring of Eastern African middle stone age populations. *Azania* **59** (1), 111–139. 10.1080/0067270X.2023.2268986 (2024).

[73] Blinkhorn, J. & Grove, M. The structure of the middle stone age of Eastern Africa. *Quat Sci. Rev.* **195**, 1–20. 10.1016/j.quascirev.2018.07.011 (2018).

[74] Tryon, C. A. The middle/later stone age transition and cultural dynamics of late pleistocene East Africa. *Evol. Anthropol.* **28**, 267–282. 10.1002/evan.21802 (2019). 31621987 10.1002/evan.21802

[75] Fusco, M. et al. The environmental context of the Middle-to-Late Stone Age Transition in eastern Africa: seasonality as a key factor. bioRxiv (2024).

[76] Loftus, E., Lee-Thorp, J., Leng, M., Marean, C. & Sealy, J. Seasonal scheduling of shellfish collection in the middle and later stone ages of Southern Africa. *J. Hum. Evol.* **128**, 1–16 (2019). 30825979 10.1016/j.jhevol.2018.12.009

[77] Linares-Matas, G. J. & Clark, J. Seasonality and Oldowan behavioral variability in East Africa. *J. Hum. Evol.* **164**, 103070 (2022). 34548178 10.1016/j.jhevol.2021.103070

[78] Leonardi, M., Hallet, E.Y., Beyer, R., Krapp, M. & Manica, A. pastclim 1.2: an R package to easily access and use paleoclimatic reconstructions. *Ecography* **2023**, e06481. 10.1111/ecog.06481 (2023).

[79] Timbrell, L. et al. More is not always better: downscaling climate model outputs from 30 to 5-minute resolution has minimal impact on coherence with late quaternary proxies. *Clim. Past*. 10.5194/cp-2024-53 (2024).

[80] Lisiecki, L. E. & Raymo, M. E. A Pliocene-Pleistocene stack of 57 globally distributed benthic δ18O records. *Paleoceanog Palaeoclimat*. **20** (1). 10.1029/2004PA001071 (2005).

[81] 81. Marwick, B. *gsloid: Global Sea Level and Oxygen Isotope Data.* CRAN R Package. Available at: https://doi.org/10.32614/CRAN.package.gsloid (2022).

[82] Massicotte, P. et al. *rnaturalearth: World Map Data from Natural Earth.* CRAN R Package. Available at: https://doi.org/10.32614/CRAN.package.rnaturalearth (2023).

