# Supplementary material for: Climate seasonality and predictability during the middle stone age and implications for technological diversification in early Homo sapiens
Source: Sci Rep. 2025 Apr 4;15:11645. doi: 10.1038/s41598-025-95573-y (PMC11971293; doi:10.1038/s41598-025-95573-y)
Supplement: Supplementary file 1 — Supplementary Material 1 [file 41598_2025_95573_MOESM1_ESM.docx]

Supplementary Online Materials: Climate seasonality and predictability during the Middle Stone Age and implications for technological diversification in early *Homo sapiens*. (L Timbrell et al)

**SOM 1. Overview of the Middle Stone Age archaeological records of eastern and northwestern Africa.**

The Middle Stone Age (MSA) has received much attention because it is becoming increasingly associated with the earliest *H. sapiens* in Africa. Starting ~300 thousand of years ago (kya) across most (Barham *et al.* 2015; Grun 2016; Hublin *et al.* 2017; Potts *et al.* 2018, 2020), but not all (Douze et al. 2021), of Africa, large cutting tools were largely abandoned in favour of prepared-core technology and the hafting of pointed pieces to form projectile weapons (McBrearty and Brooks 2000). Moreover, the MSA also marks the first widespread evidence of regionally distinctive material culture styles in hominin evolution (Clark 1982, 1988). The spatiotemporally patchy appearance of these ‘specific’ regional innovations occurs alongside more ‘generic’ elements, such as Levallois reduction and scrapers, that seem to persist across large areas throughout much of the MSA.

**SOM 1.1. Eastern Africa**

Eastern Africa, defined as the modern-day countries of Tanzania, Kenya, Uganda, Ethiopia, Somalia, Djibouti, and Eritrea (following Tryon and Faith 2013), occupies a central, equatorial position within Africa. It is unique for the continent in that it exhibits a vast mosaic of biodiversity, with combinations of woodlands, shrublands, rainforests, grasslands, and deserts occurring within relatively close proximity, in part a result of tectonic faulting which has helped create a series of variable localised landscapes (Mirazón Lahr and Foley 2016). Recent work has highlighted that, during the MSA, early *Homo sapiens* populations occupied a diverse array of ecologies in eastern Africa (see Timbrell 2024 for a recent review), such as coasts (e.g. Roberts *et al.* 2020), mountains (e.g. Groos *et al.* 2021) and ecotonal grass/shrubland (Basell 2008; Timbrell *et al.* 2022).

Paralleling the region’s palaeoecology, and indeed fossil record (Brauer *et al.* 1997; White *et al.* 2003; McDougall *et al.* 2005; Tryon *et al.* 2015; Martinón-Torres *et al.* 2021), eastern Africa’s MSA record has been described as being ‘highly variable’ (Scerri and Will, 2023; Timbrell 2024). Long periods of relative behavioural stability are also nonetheless apparent (Scerri and Will, 2023), in terms of the early and enduring appearance of bifacial points (Tryon and Faith, 2013; Blinkhorn and Grove, 2018), long-distance raw material transport in certain areas (Blegen *et al.* 2018; Brooks *et al.* 2018) pointing to extended interaction networks over large spaces and time periods (Miller and Wang 2022), and evidence for hafting, inferred from the size and shape of stone points and scrapers (Brooks *et al.* 2006; Shea 2006).

Early work by Clark (1988) pointed out that there are sites with deep stratigraphy in diverse localities that do not appear to show consistent trends through time, yet certain areas seem to have distinctive behavioural signatures, such as in the Lake Victoria Basin, the coastal region and Ethiopia. This has since been supported by work across the region. Tryon (2019) found that small point and Levallois based assemblages from the Lake Victoria Basin are quite distinct from similarly dated deposits to the west such as at the ∼60–70,000-year-old Katanda sites on the Congo/Uganda border, which yield distinctive barbed bone harpoons (Brooks *et al.* 1995). Additionally, Panga ya Saidi in the regions coastal zone has revealed a distinct cultural signal, represented by an early phase of the LSA where typical MSA Levallois cores appear alongside LSA backed artefacts and blades, as well as the first putative evidence of ostrich eggshell beads in eastern Africa (Shipton *et al.* 2018; d’Errico *et al.* 2020). Exploitation of primarily local raw materials, as well as a clear absence of obsidian, seems to be a regional character of the Ethiopian MSA, as seen at Gotera (Fusco *et al.* 2021), Melka Kunture (Mussi *et al.* 2014), Gademotta (Douze and Delagnes 2016) and Mochena Borago (Brandt *et al.* 2012). Clark (1988) originally proposed that these distinctive elements of eastern African MSA assemblages are reflective of local style as well as strategic and technological innovations, driven by the diverse topological environments and climatic conditions of the region, as well as raw material availability (Clark 1988). Clark’s (1988) hypothesis has since been proven to be robust since increased chronological resolution has improved our understanding of eastern African population dynamics (Basell 2008; Blinkhorn and Grove 2018, 2021; Timbrell *et al.* 2022).

Although such intra-regional distinctions are apparent, eastern Africa does not appear to have formally defined ‘diagnostic styles’ of MSA tools that can be used to characterise MSA industries or variants, as is seen in other regions like northern Africa (see below). Eastern African archaeologists nonetheless have tried to describe patterns of variability using terms that are usually limited to individual sites, with only a few appearing at more than one site located over 50 km apart, and/or are borrowed nomenclature from other regions, such as ‘Still Bay’ from South Africa. In a recent synthesis Shea (2020) described the current situation as ‘lithic systems anarchy’, with Timbrell (2024) suggesting that these perceived differences between the equatorial MSA record and those at more extreme latitudes may be a result of both differences in research histories as well as the cultural expressions of populations in core (refugial) versus periphery areas across the continent.

**SOM 1.2. Northwestern Africa**

Northern Africa includes the modern states of Morocco, Algeria, Tunisia, Libya, Egypt, and part of Sudan (Egyptian and Sudanese Nubia), following Ben Arous et al. (2025). Middle Pleistocene occupation of the region is documented primarily in Morocco at Jebel Irhoud, where the earliest *H. sapiens* fossil in Africa were discovered (Hublin et al. 2017; Richter et al. 2017), as well as at Wadi Lazalim in south Tunisia. From MIS 5 onwards, MSA sites are distributed from the Atlantic coast to the Red Sea coast and across the Sahara, in contrast to MIS 4-3 sites, which are much more geographically restricted. MIS 5 is associated with episodes of increased humidity, known as Green Sahara events (Drake et al., 2013), suggesting that climatic amelioration and the expansion of grasslands in the Late Pleistocene led to human occupation of newly available and largely uninhabited territories within the Sahara from sub-Saharan/non-Saharan regions (Drake *et al.* 2011; Scerri et al. 2014). During this period, the existence of lakes and rivers/wadis within the Sahara region is apparent (Drake et al. 2011), which were likely favourable environments for human and faunal populations, facilitating their dispersal along paleohydrological networks (Drake et al., 2013; Scerri et al. 2014). Indeed, population networks appear to be structured along water corridors, with more archaeological similarities than expected based on geographic proximity occurring between sites connected by rivers and lakes (Scerri et al. 2014). Aridification of the Sahara from MIS 4 likely restricted the mobility and settlement strategies of human populations towards coastal or sub-Saharan regions (Boisard et al. 2024).

In northwestern Africa, the MSA (particularly during MIS 5) includes two main lithic industries or variants: the ‘Aterian’ and the ‘Mousterian’, which overlap in time (Dibble et al. 2013; Boisard and Ben Arous, 2024). The Aterian is identified by the presence of tanged and foliated tools, dating between 150 ka and 28 ka (Ben Arous et al., 2022) and occurring from the Atlantic coast to the southern boundaries of the contemporary Sahara and sporadically documented in Cyrenaica, the Western Desert, and the Nile Valley in Egypt. In Cyrenaica, there is evidence of a systematic utilization of diverse Levallois debitage methods in Aterian assemblages, which markedly differs from practices observed in the Maghreb and the Nile Valley (Jones et al., 2016; Scerri, 2017), a pattern potentially relating to isolation, social boundaries, topography, ecology and/or water availability (Scerri 2013). The Mousterian, described as ‘Aterian lacking tanged tools’ (e.g. Dibble et al., 2013), is dated between 300 and 27 ka (Boisard and Ben Arous, 2024).

Although not the focus of our study, in northeastern Africa, MSA sites dated to MIS 5 are attributed to the ‘Nubian Complex’ (Van Peer, 1998), involving distinct lithic industries sharing several technological features, including a specific point production technique known as Nubian Levallois. The Nubian Complex, also identified in the Levant, Arabia, and eastern Africa, has been proposed as an archaeological indicator of out-of-Africa dispersals during MIS 5. However, the presence of the Nubian Levallois technique in further geographically and chronologically distant contexts, such as South Africa (Will et al. 2015) and Neanderthals in Southwest Asia (Blinkhorn et al. 2021), has raised questions about its significance for *Homo sapiens* dispersal, resulting in a lack of consensus within the scientific community (Leplongeon, 2022). Recently, Nubian Levallois reduction has been shown to be uniquely associated with arid palaeoenvironments in these diverse areas (Samawi and Hallinan 2024).

In general, these broad taxonomic entities (Mousterian, Aterian, Nubian Complex) are the product of a complex history of research in North Africa; the discovery of new sites and better dating has both broadened the definitions of these entities and triggered criticism of their use (e.g., Scerri and Spinapolice, 2019). As such, north African archaeology faces a similar ‘taxonomy’ crisis as is the case for other regions of Africa, especially south (e.g., Wilkins, 2020).

**Supplementary Tables**

Supplementary Table S1. List of Middle Stone Age occupations included in this analysis from northwestern (N) and eastern (E) Africa, with extracted mean annual temperature (bio01), temperature seasonality (bio04), total annual precipitation (bio12), precipitation seasonality (bio15) and net primary productivity (NPP) at the mid-age, as well as the percentage of temperature variability (T-V), precipitation variability (P-V) and net-primary productivity variability (NPP-V) across the date range, calculated using the change-variability decomposition algorithm.

| Occupation | R | bio01 | bio12 | bio04 | bio15 | NPP | T-V | P-V | NPP-V |
| --- | --- | --- | --- | --- | --- | --- | --- | --- | --- |
| Benzú_2 | N | 12.15 | 712.30 | 334.98 | 70.95 | 160.48 | 31.82 | 13.52 | 38.93 |
| Benzú_5 | N | 11.44 | 727.12 | 401.39 | 72.00 | 112.09 | NA | NA | NA |
| Benzú_3b | N | 11.56 | 740.56 | 505.32 | 75.25 | 117.40 | NA | NA | NA |
| Bizmoune_3 | N | 13.71 | 345.66 | 264.21 | 77.76 | 224.47 | NA | NA | NA |
| Bizmoune_2b | N | 12.72 | 445.99 | 291.34 | 81.40 | 197.40 | NA | NA | NA |
| Bizmoune_3b-3inf | N | 14.95 | 365.13 | 261.69 | 78.99 | 256.18 | 21.38 | 3.74 | 36.93 |
| Bizmoune_4a | N | 13.79 | 403.09 | 456.04 | 72.51 | 253.71 | NA | NA | NA |
| Bizmoune_4c | N | 13.71 | 345.66 | 264.21 | 77.76 | 224.47 | 5.09 | 2.83 | 6.60 |
| ChateperonRouge1 | N | 11.01 | 625.58 | 356.88 | 77.95 | 106.27 | NA | NA | NA |
| Contrebandiers_4 | N | 14.45 | 573.65 | 349.09 | 76.38 | 247.17 | NA | NA | NA |
| Contrebandiers_4d-e | N | 14.85 | 534.70 | 518.50 | 74.25 | 249.16 | NA | NA | NA |
| Contrebandiers_5 | N | 14.62 | 564.21 | 594.92 | 76.87 | 238.46 | NA | NA | NA |
| Contrebandiers_5c | V | 16.87 | 457.13 | 420.26 | 73.71 | 361.23 | 6.47 | 2.83 | 6.60 |
| Contrebandiers_5b | N | 14.85 | 534.70 | 518.50 | 74.25 | 249.16 | 24.90 | 16.07 | 19.49 |
| Contrebandiers_5a | N | 14.85 | 534.70 | 518.50 | 74.25 | 249.16 | 17.65 | 9.78 | 13.27 |
| Contrebandiers_4c | N | 13.87 | 582.60 | 579.04 | 77.24 | 211.75 | NA | NA | NA |
| Contrebandiers_4d | N | 13.33 | 615.21 | 514.96 | 79.03 | 206.52 | 13.57 | 5.62 | 10.56 |
| Contrebandiers_6c | N | 16.08 | 544.37 | 668.77 | 71.99 | 304.98 | 3.39 | 2.90 | 5.52 |
| Contrebandiers_IV-2 | N | 12.97 | 662.94 | 456.37 | 79.68 | 190.20 | 24.27 | 12.30 | 25.31 |
| Contrebandiers_IV-2a | N | 15.30 | 521.68 | 401.75 | 73.45 | 310.03 | NA | NA | NA |
| Contrebandiers_IV-2b | N | 14.69 | 541.62 | 545.74 | 74.48 | 243.65 | NA | NA | NA |
| Contrebandiers_V-1b | N | 15.60 | 507.43 | 361.48 | 75.92 | 308.61 | NA | NA | NA |
| Contrebandiers_V-2 | N | 13.51 | 605.67 | 547.41 | 78.56 | 208.75 | NA | NA | NA |
| Dar_es-Soltan1_Group1_G1.10 | N | 16.18 | 559.35 | 655.42 | 73.41 | 312.95 | NA | NA | NA |
| Dar_es-Soltan1_Group1_G1.7 | N | 15.04 | 533.78 | 386.04 | 76.71 | 272.03 | NA | NA | NA |
| Dar_es-Soltan1_Group3_G3.11 | N | 13.70 | 544.27 | 452.28 | 75.76 | 219.33 | NA | NA | NA |
| Dar_es-Soltan1_Group1_G1.8 | N | 16.18 | 559.35 | 655.42 | 73.41 | 312.95 | NA | NA | NA |
| Dar_es-Soltan1_Group2_G2.1 | N | 15.60 | 507.43 | 361.48 | 75.92 | 308.61 | NA | NA | NA |
| Dar_es-Soltan1_Group2_G2.6 | N | 13.91 | 588.38 | 449.82 | 78.34 | 226.20 | NA | NA | NA |
| Dar_es-Soltan1_Group3_G3.1 | N | 13.52 | 634.87 | 406.74 | 78.78 | 206.38 | NA | NA | NA |
| Dar_es-Soltan1_Group3_G3.3 | N | 13.29 | 646.48 | 491.58 | 79.17 | 200.35 | NA | NA | NA |
| Dar_es-Soltan1_Group3_G3.4 | N | 14.16 | 589.30 | 563.79 | 75.94 | 231.03 | NA | NA | NA |
| Dar_es-Soltan1_Group4_G4.7 | N | 13.64 | 589.76 | 448.90 | 76.28 | 226.57 | NA | NA | NA |
| Dar_es-Soltan2_5 | N | 14.29 | 554.43 | 550.97 | 75.88 | 225.16 | 0.00 | 0.00 | 0.00 |
| Dar_es-Soltan2_7 | N | 16.74 | 485.95 | 581.38 | 72.88 | 327.22 | NA | NA | NA |
| El_Harhoura1_1 | N | 11.01 | 648.64 | 395.46 | 79.09 | 108.59 | NA | NA | NA |
| El_Harhoura1_2 | N | 12.03 | 660.88 | 359.59 | 78.80 | 137.96 | NA | NA | NA |
| El_Harhoura2_3 | N | 12.40 | 645.80 | 359.40 | 78.05 | 145.78 | NA | NA | NA |
| El_Harhoura2_8 | N | 14.45 | 573.65 | 349.09 | 76.38 | 247.17 | NA | NA | NA |
| El_Harhoura2_9 | N | 13.33 | 615.21 | 514.96 | 79.03 | 206.52 | NA | NA | NA |
| El_Harhoura2_4b | N | 13.01 | 619.42 | 377.54 | 76.97 | 193.11 | NA | NA | NA |
| El_Harhoura2_4a | N | 12.74 | 628.55 | 375.79 | 77.00 | 169.78 | NA | NA | NA |
| El_Mnasra_8a | N | 13.88 | 613.20 | 383.81 | 78.01 | 228.32 | 29.85 | 16.60 | 21.91 |
| El_Mnasra_8b | N | 15.01 | 539.10 | 354.99 | 74.85 | 301.06 | 27.18 | 23.12 | 21.76 |
| El_Mnasra_8c-d | N | 14.45 | 573.65 | 349.09 | 76.38 | 247.17 | 22.05 | 18.40 | 17.61 |
| El_Mnasra_9 | N | 13.88 | 613.20 | 383.81 | 78.01 | 228.32 | NA | NA | NA |
| El_Mnasra_9c | N | 13.71 | 600.37 | 488.75 | 78.69 | 219.83 | NA | NA | NA |
| El_Mnasra_4 | N | 11.44 | 656.99 | 353.16 | 79.69 | 135.79 | NA | NA | NA |
| El_Mnasra_6 | N | 13.51 | 591.28 | 488.43 | 77.53 | 199.53 | 6.15 | 4.34 | 8.21 |
| El_Mnasra_7 | N | 14.85 | 534.70 | 518.50 | 74.25 | 249.16 | 19.62 | 10.18 | 13.40 |
| Haua_Fteah_498 | N | 15.49 | 355.47 | 537.95 | 94.65 | 50.52 | NA | NA | NA |
| Haua_Fteah_513 | N | 15.46 | 353.63 | 539.33 | 94.44 | 50.78 | NA | NA | NA |
| Haua_Fteah_524 | N | 14.54 | 341.40 | 538.24 | 91.28 | 46.20 | NA | NA | NA |
| Haua_Fteah_528 | N | 15.76 | 327.30 | 498.22 | 95.42 | 57.03 | NA | NA | NA |
| Haua_Fteah_752 | N | 14.12 | 361.89 | 592.82 | 89.06 | 46.36 | 0.00 | 0.00 | 0.00 |
| Haua_Fteah_770 | N | 16.42 | 381.41 | 600.11 | 91.66 | 66.67 | NA | NA | NA |
| Haua_Fteah_776 | N | 16.08 | 384.07 | 556.62 | 94.39 | 59.04 | NA | NA | NA |
| Haua_Fteah_798 | N | 16.32 | 370.63 | 585.13 | 92.20 | 65.14 | NA | NA | NA |
| Haua_Fteah_811 | N | 18.31 | 340.23 | 475.02 | 102.13 | 79.39 | NA | NA | NA |
| Haua_Fteah_817 | N | 17.83 | 431.26 | 625.25 | 92.55 | 77.85 | NA | NA | NA |
| Haua_Fteah_824 | N | 18.31 | 340.23 | 475.02 | 102.13 | 79.39 | NA | NA | NA |
| Haua_Fteah_825 | N | 17.94 | 419.74 | 613.96 | 93.20 | 85.51 | NA | NA | NA |
| Haua_Fteah_827 | N | 17.80 | 441.05 | 630.81 | 92.31 | 71.60 | NA | NA | NA |
| Haua_Fteah_828 | N | 18.49 | 369.00 | 531.56 | 98.65 | 89.00 | NA | NA | NA |
| Haua_Fteah_832 | N | 16.52 | 426.58 | 614.94 | 91.52 | 70.18 | 2.94 | 0.57 | 10.25 |
| Haua_Fteah_837 | N | 14.24 | 346.49 | 577.78 | 89.70 | 47.54 | NA | NA | NA |
| Haua_Fteah_503-504 | N | 15.33 | 353.05 | 538.28 | 94.34 | 48.99 | NA | NA | NA |
| Haua_Fteah_525-565 | N | 16.36 | 329.41 | 493.76 | 96.88 | 63.17 | NA | NA | NA |
| Haua_Fteah_536-537-562 | N | 15.14 | 326.94 | 505.83 | 93.85 | 50.87 | NA | NA | NA |
| IfrinAmmar_UnitD | N | 13.12 | 450.88 | 342.96 | 62.85 | 173.59 | 21.98 | 10.69 | 31.58 |
| IfrinAmmar_XV | N | 14.37 | 493.63 | 661.84 | 68.01 | 193.11 | NA | NA | NA |
| IfrinAmmar_XVII | N | 15.10 | 514.97 | 702.34 | 67.20 | 206.18 | NA | NA | NA |
| IfrinAmmar_XXII | N | 11.30 | 516.67 | 444.34 | 69.04 | 114.17 | NA | NA | NA |
| IfrinAmmar_XXXI | N | 11.69 | 560.97 | 461.62 | 68.87 | 123.05 | 10.53 | 6.13 | 17.13 |
| JebelIrhoud_4 | N | 16.55 | 384.37 | 638.09 | 65.29 | 294.04 | 7.72 | 4.36 | 10.54 |
| JebelIrhoud_5 | N | 14.91 | 353.38 | 405.66 | 71.66 | 253.49 | 23.87 | 8.98 | 27.99 |
| JebelIrhoud_6 | N | 14.75 | 369.77 | 334.51 | 73.69 | 257.93 | 21.58 | 6.51 | 21.82 |
| JebelIrhoud_7 | N | 12.49 | 514.09 | 397.88 | 80.58 | 213.56 | 15.74 | 10.79 | 10.18 |
| Oued_el_Akarit_S-8(A) | N | 16.96 | 168.94 | 870.40 | 63.74 | 139.69 | NA | NA | NA |
| Oued_el_Akarit_S-8-EU-8006 | N | 16.51 | 169.71 | 853.94 | 63.59 | 128.49 | NA | NA | NA |
| Rhafas_3 | N | 12.98 | 416.26 | 704.05 | 60.57 | 170.21 | NA | NA | NA |
| Rhafas_3a | N | 12.96 | 422.62 | 475.11 | 56.48 | 165.77 | 48.61 | 7.04 | 39.51 |
| Rhafas_3b | N | 13.18 | 414.92 | 457.73 | 55.30 | 173.47 | 33.93 | 6.86 | 37.09 |
| Rhafas_4c | N | 11.87 | 448.04 | 358.53 | 59.47 | 148.64 | NA | NA | NA |
| Rhafas_6d | N | 12.64 | 412.61 | 711.32 | 58.81 | 159.22 | 3.55 | 1.50 | 5.33 |
| Rhafas_S5 | N | 11.74 | 435.38 | 594.16 | 62.01 | 132.88 | NA | NA | NA |
| Rhafas_S6 | N | 12.05 | 451.92 | 600.54 | 59.72 | 149.28 | NA | NA | NA |
| Rhafas_S7 | N | 15.84 | 378.07 | 801.13 | 62.70 | 252.90 | NA | NA | NA |
| ShakshukWest | N | 17.36 | 126.90 | 835.38 | 78.56 | 107.26 | NA | NA | NA |
| Taforalt_180-194 LH_C | N | 10.81 | 444.46 | 412.83 | 67.05 | 92.71 | NA | NA | NA |
| Taforalt_210-224 LH_C | N | 11.75 | 462.07 | 468.61 | 65.43 | 131.51 | NA | NA | NA |
| Taforalt_Calcareous_Group | N | 15.67 | 345.52 | 494.13 | 54.17 | 246.06 | 4.58 | 2.52 | 10.03 |
| Taforalt_Group_C | N | 10.49 | 457.20 | 425.29 | 67.53 | 92.60 | NA | NA | NA |
| Taforalt_Group_D | N | 11.94 | 464.60 | 515.01 | 62.32 | 124.22 | NA | NA | NA |
| Taforalt_Group_E | N | 12.80 | 381.80 | 437.11 | 59.22 | 149.58 | 15.92 | 3.87 | 45.50 |
| Taforalt_Group_F | N | 14.24 | 407.26 | 488.63 | 56.58 | 200.12 | 33.09 | 17.04 | 49.14 |
| Taforalt_Y12 | N | 10.95 | 462.39 | 454.75 | 66.69 | 109.04 | NA | NA | NA |
| Taforalt_Y4 | N | 10.18 | 457.78 | 324.46 | 64.59 | 77.98 | NA | NA | NA |
| Taforalt_Y5 | N | 10.18 | 457.78 | 324.46 | 64.59 | 77.98 | NA | NA | NA |
| Taforalt_Y6 | N | 10.62 | 446.90 | 390.96 | 66.69 | 87.40 | NA | NA | NA |
| Taforalt_Y7 | N | 10.49 | 457.20 | 425.29 | 67.53 | 92.60 | NA | NA | NA |
| Uan_Afuda_dune | N | 20.08 | 10.46 | 638.07 | 118.74 | 19.21 | NA | NA | NA |
| Uan_Tabu_22 | N | 18.02 | 22.14 | 867.98 | 124.92 | 25.78 | NA | NA | NA |
| Wadi_Lazalim_15/1_5 | N | 18.47 | 129.22 | 669.03 | 59.13 | 155.98 | 13.17 | 2.52 | 8.00 |
| Wadi_Lazalim_15/1_9/10 | N | 18.20 | 139.76 | 702.71 | 68.46 | 132.20 | 10.15 | 6.82 | 8.00 |
| Wadi_Lazalim_15/1_4a | N | 17.52 | 143.14 | 751.36 | 65.61 | 119.33 | 44.33 | 9.78 | 18.97 |
| Wadi_Lazalim_15/1_4b | N | 17.96 | 128.51 | 600.83 | 59.40 | 145.17 | 17.48 | 3.22 | 6.53 |
| Wadi_Lazalim_16/15_G | N | 15.97 | 141.07 | 824.30 | 65.10 | 113.41 | 5.94 | 13.55 | 6.12 |
| Wadi_Lazalim_16/29_3 | N | 15.77 | 142.02 | 862.97 | 63.86 | 111.81 | 5.18 | 3.10 | 1.21 |
| Wadi_Lazalim_16/29_5 | N | 18.55 | 158.12 | 981.26 | 68.34 | 134.01 | 3.28 | 0.14 | 1.31 |
| Wadi_Lazalim_16/29_6 | N | 15.78 | 143.35 | 713.66 | 63.39 | 99.60 | 10.72 | 2.89 | 3.24 |
| Abdur_N_C_S | E | 25.36 | 611.34 | 430.06 | 157.44 | 405.01 | NA | NA | NA |
| AdumaA1 | E | 23.15 | 581.75 | 314.66 | 85.46 | 405.44 | NA | NA | NA |
| AdumaA4C | E | 23.15 | 581.75 | 314.66 | 85.46 | 405.44 | NA | NA | NA |
| AdumaA5Ex | E | 23.15 | 581.75 | 314.66 | 85.46 | 405.44 | NA | NA | NA |
| AdumaA5ExSurf | E | 23.15 | 581.75 | 314.66 | 85.46 | 405.44 | NA | NA | NA |
| AdumaA8 | E | 23.15 | 581.75 | 314.66 | 85.46 | 405.44 | NA | NA | NA |
| AdumaA8AC | E | 23.15 | 581.75 | 314.66 | 85.46 | 405.44 | NA | NA | NA |
| AdumaA8AG | E | 23.15 | 581.75 | 314.66 | 85.46 | 405.44 | NA | NA | NA |
| AdumaA8ASurf | E | 23.15 | 581.75 | 314.66 | 85.46 | 405.44 | NA | NA | NA |
| AdumaA8B | E | 23.15 | 581.75 | 314.66 | 85.46 | 405.44 | NA | NA | NA |
| AdumaVP1/1 | E | 23.15 | 581.75 | 314.66 | 85.46 | 405.44 | NA | NA | NA |
| AdumaVP1/3 | E | 23.15 | 581.75 | 314.66 | 85.46 | 405.44 | NA | NA | NA |
| Dorothy Garrod Site | E | 16.96 | 728.15 | 81.44 | 72.27 | 526.15 | 37.71 | 4.23 | 12.64 |
| Enkapune_ya_Muto_RBL4 | E | 13.35 | 1116.92 | 70.26 | 42.39 | 708.33 | NA | NA | NA |
| EyasiShore_77_81 | E | 19.25 | 731.73 | 123.74 | 94.48 | 547.82 | 56.16 | 4.36 | 18.69 |
| EyasiShore_N_surface | E | 19.25 | 731.73 | 123.74 | 94.48 | 547.82 | 56.16 | 4.36 | 18.69 |
| EyasiShore_W_insitu | E | 19.25 | 731.73 | 123.74 | 94.48 | 547.82 | 56.16 | 4.36 | 18.69 |
| EyasiShore_W_surf | E | 19.25 | 731.73 | 123.74 | 94.48 | 547.82 | 56.16 | 4.36 | 18.69 |
| Fincha Habera 8_10 | E | 9.70 | 1177.14 | 169.88 | 63.94 | 587.34 | NA | NA | NA |
| Fincha Habera 8_11 | E | 9.70 | 1177.14 | 169.88 | 63.94 | 587.34 | NA | NA | NA |
| Fincha Habera 8_8 | E | 9.70 | 1177.14 | 169.88 | 63.94 | 587.34 | NA | NA | NA |
| Fincha Habera 8_9 | E | 9.70 | 1177.14 | 169.88 | 63.94 | 587.34 | NA | NA | NA |
| Fincha Habera 9 | E | 10.98 | 1172.64 | 167.25 | 63.67 | 691.67 | NA | NA | NA |
| Gademotta_ETH72_1 | E | 12.01 | 1369.53 | 244.01 | 81.13 | 695.27 | 17.38 | 0.74 | 23.27 |
| Gademotta_ETH72_6 | E | 12.01 | 1369.53 | 244.01 | 81.13 | 695.27 | 17.38 | 0.74 | 23.27 |
| Goda Buticha_Complex2_DEF | E | 19.27 | 696.24 | 264.95 | 75.49 | 572.16 | 38.38 | 38.38 | 33.43 |
| Halibee_Faro Daba | E | 19.97 | 842.18 | 367.78 | 88.16 | 699.52 | 2.10 | 0.76 | 6.68 |
| KapedoTuffs | E | 22.34 | 754.32 | 126.90 | 74.64 | 759.08 | NA | NA | NA |
| KapForm_KoimilotGnJh74_1 | E | 17.82 | 1055.47 | 124.33 | 64.06 | 888.66 | 27.25 | 10.02 | 9.13 |
| KapForm_KoimilotGnJh74_2 | E | 17.82 | 1055.47 | 124.33 | 64.06 | 888.66 | 27.25 | 10.02 | 9.13 |
| KapForm_SSRS | E | 17.82 | 1055.47 | 124.33 | 64.06 | 888.66 | 27.25 | 10.02 | 9.13 |
| Karungu_A1N | E | 16.82 | 1553.19 | 83.92 | 38.05 | 1153.91 | 46.91 | 3.06 | 41.98 |
| Karungu_A2N | E | 16.82 | 1553.19 | 83.92 | 38.05 | 1153.91 | 46.91 | 3.06 | 41.98 |
| Karungu_A3Ex | E | 16.82 | 1553.19 | 83.92 | 38.05 | 1153.91 | 46.91 | 3.06 | 41.98 |
| Karungu_Kisaaka_Main | E | 15.05 | 1680.66 | 63.23 | 34.83 | 951.06 | NA | NA | NA |
| Karungu_Kisaaka_ZTG | E | 16.82 | 1553.19 | 83.92 | 38.05 | 1153.91 | 46.91 | 3.06 | 41.98 |
| Kisese II_18 | E | 15.68 | 866.63 | 97.65 | 79.37 | 430.21 | NA | NA | NA |
| Kisese II_19 | E | 15.68 | 866.63 | 97.65 | 79.37 | 430.21 | NA | NA | NA |
| Kisese II_20 | E | 15.72 | 930.62 | 139.37 | 80.38 | 453.31 | NA | NA | NA |
| Kisese II_21 | E | 16.20 | 913.31 | 145.15 | 81.64 | 554.34 | NA | NA | NA |
| Koobi_GaJj17 | E | 23.02 | 391.18 | 68.30 | 69.98 | 307.65 | NA | NA | NA |
| LaasGeel_SU_711 | E | 18.88 | 377.60 | 381.95 | 75.35 | 354.95 | NA | NA | NA |
| LukenyaHill_GvJm46 | E | 13.89 | 1052.96 | 112.20 | 73.05 | 577.24 | NA | NA | NA |
| LukenyaHillGvJm22_F170_205 | E | 14.70 | 919.23 | 112.22 | 76.61 | 552.22 | NA | NA | NA |
| Mabubike_100 to 110¬†cm | E | 16.80 | 943.35 | 167.75 | 89.05 | 622.05 | 48.88 | 25.81 | 11.50 |
| Mabubike_110 to 130¬†cm | E | 16.80 | 943.35 | 167.75 | 89.05 | 622.05 | 48.88 | 25.81 | 11.50 |
| Mabubike_130 to 140¬†cm | E | 16.80 | 943.35 | 167.75 | 89.05 | 622.05 | 48.88 | 25.81 | 11.50 |
| Mabubike_140 to 150¬†cm | E | 16.80 | 943.35 | 167.75 | 89.05 | 622.05 | 48.88 | 25.81 | 11.50 |
| Mabubike_150 to 160¬†cm | E | 16.80 | 943.35 | 167.75 | 89.05 | 622.05 | 48.88 | 25.81 | 11.50 |
| Mabubike_160 to 170 | E | 16.28 | 930.85 | 96.17 | 90.50 | 484.16 | NA | NA | NA |
| Mabubike_170 to 180¬†cm | E | 16.28 | 930.85 | 96.17 | 90.50 | 484.16 | NA | NA | NA |
| Mabubike_180 to 200¬†cm | E | 16.28 | 930.85 | 96.17 | 90.50 | 484.16 | NA | NA | NA |
| Mabubike_50 to 60¬†cm | E | 16.71 | 937.58 | 170.56 | 91.61 | 611.31 | NA | NA | NA |
| Mabubike_60 to 70¬†cm | E | 16.71 | 937.58 | 170.56 | 91.61 | 611.31 | NA | NA | NA |
| Mabubike_70 to 80¬†cm | E | 16.71 | 937.58 | 170.56 | 91.61 | 611.31 | NA | NA | NA |
| Mabubike_80 to 90¬†cm | E | 16.80 | 943.35 | 167.75 | 89.05 | 622.05 | 48.88 | 25.81 | 11.50 |
| Mabubike_90 to 100¬†cm | E | 16.80 | 943.35 | 167.75 | 89.05 | 622.05 | 48.88 | 25.81 | 11.50 |
| Magubike_MSA | E | 16.17 | 949.02 | 143.24 | 90.05 | 579.68 | NA | NA | NA |
| MalewaGorge | E | 14.50 | 1037.08 | 81.39 | 61.89 | 864.10 | NA | NA | NA |
| Marmonet Drift_H2 | E | 10.39 | 1213.76 | 92.62 | 53.51 | 495.13 | 5.03 | 2.99 | 4.50 |
| Marmonet Drift_H4 | E | 14.04 | 1050.66 | 145.94 | 66.37 | 817.46 | 63.55 | 6.14 | 15.46 |
| Marmonet Drift_H5 | E | 13.11 | 1284.58 | 126.94 | 45.77 | 805.67 | NA | NA | NA |
| Marmonet Drift_I_bottom | E | 12.09 | 1478.71 | 137.92 | 44.99 | 747.04 | 19.55 | 4.57 | 20.39 |
| MochenaBorago_LowerT | E | 14.58 | 1311.88 | 64.05 | 58.13 | 1022.22 | NA | NA | NA |
| MochenaBorago_RGroup | E | 13.92 | 1327.17 | 103.72 | 57.67 | 943.77 | NA | NA | NA |
| MochenaBorago_SGroup | E | 14.96 | 1300.08 | 75.07 | 54.25 | 1082.90 | NA | NA | NA |
| MochenaBorago_UpperT | E | 14.68 | 1304.00 | 62.82 | 55.80 | 1043.01 | NA | NA | NA |
| Moricho_GqJh3-West | E | 17.41 | 1121.96 | 71.52 | 59.52 | 932.98 | 4.71 | 4.25 | 21.07 |
| Moricho_GqJh20 | E | 11.57 | 1473.01 | 79.11 | 30.27 | 732.61 | 17.37 | 6.74 | 12.21 |
| Mumba_L_III_38 | E | 15.72 | 824.70 | 73.63 | 75.41 | 529.77 | NA | NA | NA |
| Mumba_L_VI_A | E | 17.86 | 814.69 | 186.81 | 75.29 | 525.40 | 51.26 | 0.83 | 7.18 |
| Mumba_U_VI_A | E | 15.39 | 828.25 | 67.12 | 74.56 | 544.24 | NA | NA | NA |
| Mumba_VI_B | E | 19.12 | 898.67 | 219.99 | 82.01 | 883.36 | 4.44 | 3.19 | 19.27 |
| Nasera_12_17 | E | 15.67 | 826.72 | 108.84 | 61.38 | 606.72 | NA | NA | NA |
| Nasera_6_7 | E | 16.36 | 818.59 | 116.57 | 62.97 | 590.66 | NA | NA | NA |
| Nasera_8/9_11 | E | 16.36 | 818.59 | 116.57 | 62.97 | 590.66 | NA | NA | NA |
| Ndutu_14 | E | 17.82 | 735.78 | 74.81 | 77.80 | 454.87 | 38.19 | 4.25 | 10.14 |
| Ndutu_72 | E | 17.82 | 735.78 | 74.81 | 77.80 | 454.87 | 38.19 | 4.25 | 10.14 |
| Olorgesailie_BOK1E | E | 16.34 | 816.45 | 152.22 | 59.63 | 680.76 | 10.04 | 0.77 | 12.75 |
| Olorgesailie_BOK2 | E | 16.34 | 816.45 | 152.22 | 59.63 | 680.76 | 5.88 | 0.41 | 7.72 |
| Olorgesailie_BOK3 | E | 16.34 | 816.45 | 152.22 | 59.63 | 680.76 | 5.88 | 0.41 | 7.72 |
| Olorgesailie_BOK4 | E | 15.81 | 714.89 | 89.97 | 73.54 | 626.75 | NA | NA | NA |
| Omo_AHS1-5 | E | 23.37 | 557.51 | 178.89 | 95.35 | 370.36 | NA | NA | NA |
| Omo_AHS6_8 | E | 23.37 | 557.51 | 178.89 | 95.35 | 370.36 | NA | NA | NA |
| Omo_AHSsurface | E | 23.37 | 557.51 | 178.89 | 95.35 | 370.36 | NA | NA | NA |
| Omo_BNS_L3 | E | 24.69 | 508.80 | 155.28 | 86.87 | 377.98 | NA | NA | NA |
| Omo_BNS>50m | E | 24.69 | 508.80 | 155.28 | 86.87 | 377.98 | NA | NA | NA |
| Omo_BNS<50m | E | 24.69 | 508.80 | 155.28 | 86.87 | 377.98 | NA | NA | NA |
| Omo_KHS2/3 | E | 23.37 | 557.51 | 178.89 | 95.35 | 370.36 | NA | NA | NA |
| Omo_KHSNgully | E | 23.37 | 557.51 | 178.89 | 95.35 | 370.36 | NA | NA | NA |
| Omo_KHSNMKenya | E | 23.37 | 557.51 | 178.89 | 95.35 | 370.36 | NA | NA | NA |
| Omo_KHSEgully | E | 23.37 | 557.51 | 178.89 | 95.35 | 370.36 | NA | NA | NA |
| Omo_KH_surface | E | 23.37 | 557.51 | 178.89 | 95.35 | 370.36 | NA | NA | NA |
| Omo_KHSSgully | E | 23.37 | 557.51 | 178.89 | 95.35 | 370.36 | NA | NA | NA |
| Pange_ya_Saidi_17 | E | 23.38 | 1088.32 | 144.73 | 52.04 | 1060.08 | NA | NA | NA |
| Pange_ya_Saidi_18 | E | 23.29 | 940.18 | 144.61 | 64.03 | 671.43 | NA | NA | NA |
| Pange_ya_Saidi_19 | E | 23.29 | 940.18 | 144.61 | 64.03 | 671.43 | NA | NA | NA |
| PorcEpic_III | E | 17.72 | 727.94 | 338.78 | 80.12 | 542.37 | NA | NA | NA |
| PorcEpic_IV | E | 18.07 | 702.23 | 290.51 | 76.00 | 549.84 | NA | NA | NA |
| Rusinga_Nyamita | E | 16.09 | 1718.12 | 57.51 | 33.67 | 1094.98 | NA | NA | NA |
| Shinfa-Metema 1 | E | 22.59 | 995.45 | 192.07 | 130.25 | 769.13 | NA | NA | NA |
| Shurmai_MSA | E | 16.22 | 1024.34 | 73.48 | 75.58 | 778.89 | NA | NA | NA |
| Simbi | E | 16.99 | 1227.65 | 94.39 | 54.41 | 993.76 | 5.91 | 4.19 | 11.45 |
| Singa | E | 26.92 | 621.36 | 265.71 | 102.08 | 421.34 | 8.08 | 3.53 | 10.19 |
| VictoriaCabera_2 | E | 17.45 | 705.59 | 96.81 | 63.92 | 487.08 | NA | NA | NA |
| VictoriaCabera_2a | E | 17.45 | 705.59 | 96.81 | 63.92 | 487.08 | NA | NA | NA |
| VictoriaCabera_3 | E | 17.45 | 705.59 | 96.81 | 63.92 | 487.08 | NA | NA | NA |
| VictoriaCabera_4 | E | 17.69 | 666.13 | 63.06 | 73.58 | 462.40 | NA | NA | NA |
| VictoriaCabera_5 | E | 17.69 | 666.13 | 63.06 | 73.58 | 462.40 | NA | NA | NA |
| VictoriaCabera_5a | E | 17.69 | 666.13 | 63.06 | 73.58 | 462.40 | NA | NA | NA |
| Ziway-Shala_B1s3 | E | 11.36 | 1211.59 | 130.47 | 76.25 | 620.03 | NA | NA | NA |
| Ziway-Shala_DW1 | E | 11.56 | 1228.59 | 152.00 | 77.77 | 605.43 | NA | NA | NA |

Supplementary Table S2. Results from the permuted Mann-Whitney U tests comparing average mean annual temperature (bio01), temperature seasonality (bio04), total annual precipitation (bio12), precipitation seasonality (bio15) and net primary productivity (NPP) between northwestern and eastern African Middle Stone Age occupations. Robust results are those where the W coefficient produced by the mid-age is similar to the median from the permuted distribution, falling into the centre of the distribution. Unreliable results are those that fall within the 0.05 or 0.95 percentile of the distribution and are marked by *.

|  | Original coefficient using mid-age | Median coefficient of permuted distribution | Percentile of distribution corresponding to the original coefficient |
| --- | --- | --- | --- |
| Bio01 | 4425 | 4452 | 0.41 |
| Bio12 | 5954 | 5901 | 0.88 |
| Bio04 | 72 | 84 | 0.58 |
| Bio15 | 2689 | 2645 | 0.44 |
| NPP | 6246 | 6243 | 0.73 |

Supplementary Table S3. Results from the permuted Ansari-Bradley tests comparing variability of mean annual temperature (bio01), temperature seasonality (bio04), total annual precipitation (bio12), precipitation seasonality (bio15) and net primary productivity (NPP) between northwestern and eastern African Middle Stone Age occupations. Robust results are those where the W coefficient produced by the mid-age is similar to the median from the permuted distribution, falling into the centre of the distribution. Unreliable results are those that fall within the 0.05 or 0.95 percentile of the distribution and are marked by *.

|  | Original coefficient using mid-age | Median coefficient of permuted distribution | Percentile of distribution corresponding to the original coefficient |
| --- | --- | --- | --- |
| Bio01 | 2137 | 2210.5 | 0.16 |
| Bio12 | 1822 | 1782 | 0.89 |
| Bio04 | 1824 | 1835 | 0.59 |
| Bio15 | 2159 | 2048 | 0.98* |
| NPP | 1778 | 1781 | 0.35 |

**Supplementary Figures**


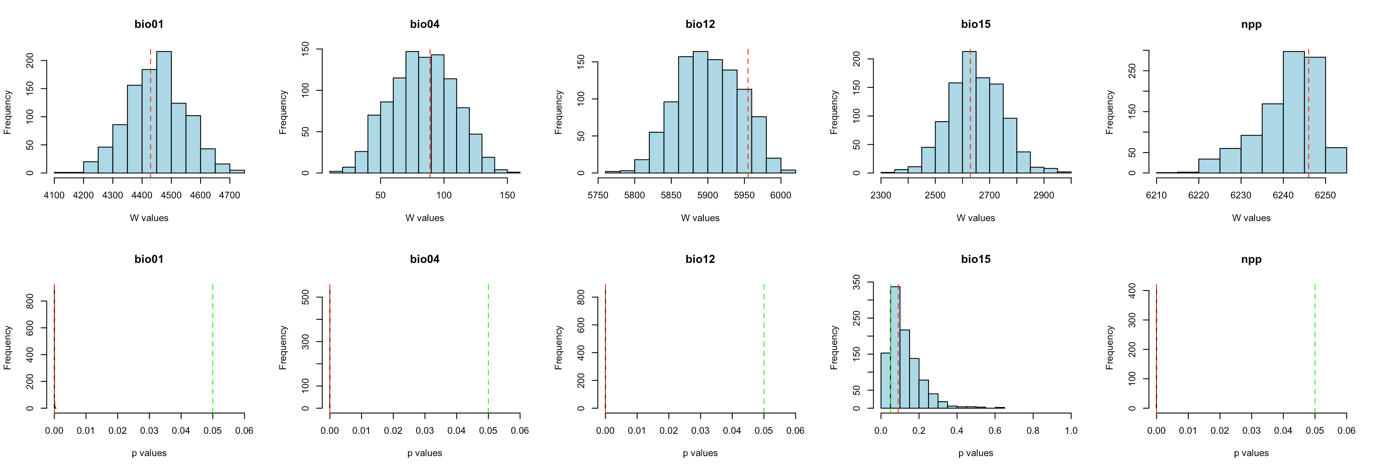


Supplementary Figure S1. Histograms of *W* values and associated p-values based on permutations across the date range of each occupation with that of the mid-age highlighted by a red dashed line. Alpha level of 0.05 is marked by a green dashed line.


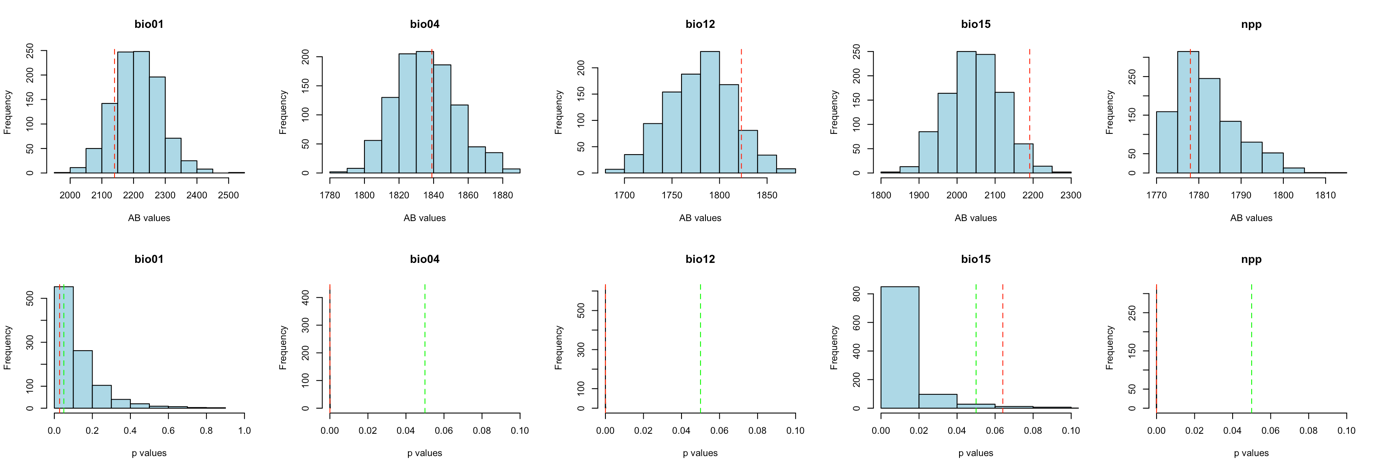


Supplementary Figure S2. Histograms of *AB* values and associated p-values based on permutations across the date range of each occupation with that of the mid-age highlighted by a red dashed line. Alpha level of 0.05 is marked by a green dashed line.


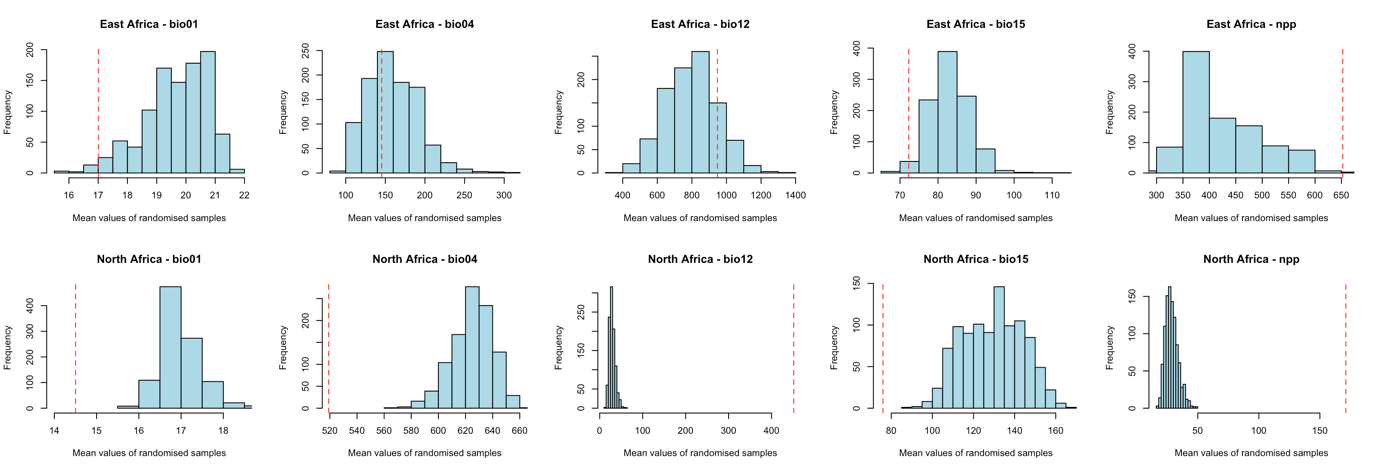


Supplementary Figure S3. Histograms of median climatic values based on random samples (1000 permutations) across the region, with that of the actual Middle Stone Age occupations highlighted by a red dashed line.


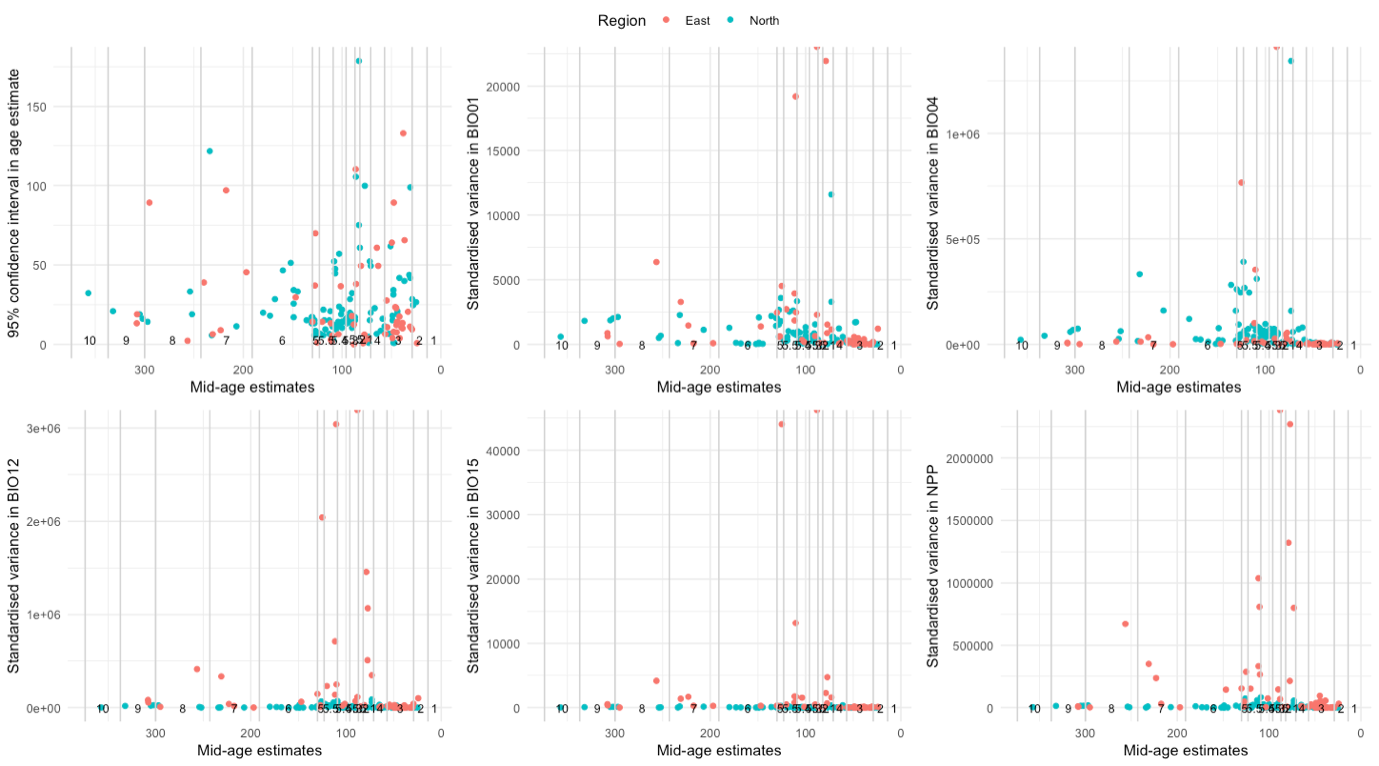


Supplementary Figure S4. Variance in the climatic variables standardised by the 95% confidence interval of dating uncertainty in relation to the mid-age estimate of the occupations.

**References:**

Barham, L., Tooth, S., Duller, G. A., Plater, A. J. & Turner, S. Excavations at Site C North, Kalambo Falls, Zambia: New insights into the mode 2/3 transition in south-central Africa. *J. Afr. Archaeol.* **13**, 187–214 (2015).

Basell, L. S. Middle Stone Age (MSA) site distributions in eastern Africa and their relationship to Quaternary environmental change, refugia and the evolution of Homo sapiens. *Quat. Sci. Rev.* **27**, 2484–2498 (2008).

Ben Arous, E. *et al.* An improved chronology for the Middle Stone Age at El Mnasra cave, Morocco. *PLoS ONE* **17**, e0261282 (2022).

Ben Arous, E., Boisard, S. & Leplongeon, A. The Upper Pleistocene Archaeology of northern Africa (Middle and Later Stone Age, from the western Maghreb to the Nile Valley). In *Encyclopedia of Quaternary Science*, 3rd edn (ed. Elias, S.) vol. 1, 108–122 (Elsevier, 2025).

Blegen, N., Jicha, B. & McBrearty, S. A new tephrochronology for early diverse stone tool technologies and long-distance raw material transport in the Middle to Late Pleistocene Kapthurin Formation, East Africa. *J. Hum. Evol.* **121**, (2018).

Blinkhorn, J. & Grove, M. The structure of the Middle Stone Age of eastern Africa. *Quat. Sci. Rev.* **195**, 1–20 (2018).

Blinkhorn, J. & Grove, M. Explanations of variability in Middle Stone Age stone tool assemblage composition and raw material use in Eastern Africa. *Archaeol. Anthropol. Sci.* **13**, 14 (2021).

Blinkhorn, J. *et al.* Nubian Levallois technology associated with southernmost Neanderthals. *Sci. Rep.* **11**, 2869 (2021).

Boisard, S. & Ben Arous, E. A Critical Inventory and Associated Chronology of the Middle Stone Age and Later Stone Age in Northwest Africa. *J. Open Archaeol. Data* **12**, 5 (2024).

Boisard, S., Wren, C., Timbrell, L. & Burke, A. Climate frameworks for the Middle Stone Age and Later Stone Age in Northwest Africa. *Quat. Int.* **716**, 109593 (2025).

Brandt, S., Fisher, E. C., Hildebrand, E. A., Vogelsang, R., Ambrose, A. H., Lesur, J. & Wang, H. Early MIS 3 occupation of Mochena Borago Rockshelter, southwest Ethiopian Highlands: implications for Late Pleistocene archaeology, paleoenvironments and modern human dispersals. *Quat. Int.* **274**, 38–54 (2012).

Brauer, G., Yokoyama, Y., Falguères, C. & Mbua, E. Modern human origins back-dated. *Nature* **386**, 337–338 (1997).

Brooks, A. S. et al.. Dating and context of three Middle Stone Age sites with bone points in the upper Semliki Valley, Zaire. *Science* **268**, 548–553 (1995).

Brooks, A. S., Yellen, J. E., Nevell, L. & Hartman, G. Projectile technologies of the African MSA: Implications for modern human origins. In *Transitions Before the Transition: Evolution and Stability in the Middle Paleolithic and Middle Stone Age* (eds. Hovers, E. & Kuhn, S. L.) 233–256 (Springer, 2006).

Brooks, A. S. et al. Long-distance stone transport and pigment use in the earliest Middle Stone Age. *Science* **360**, 90–94 (2018).

Clark, J. D. *The Cultures of the Middle Palaeolithic/Middle Stone Age*. (Cambridge Univ. Press, 1982).

Clark, J. D. The Middle Stone Age of East Africa and the beginnings of regional identity. *J. World Prehist.* **2**, 235–305 (1988).

d’Errico, F., Marti, A. P., Shipton, C., Le Vraux, E., Ndiema, E., Goldstein, S., Petraglia, M. & Boivin, N. Trajectories of cultural innovation from the Middle to Later Stone Age in Eastern Africa: personal ornaments, bone artifacts, and ochre from Panga ya Saidi, Kenya. *J. Hum. Evol.* **141**, 102737 (2020).

Dibble, H. L., Aldeias, V., Jacobs, Z., Olszewski, D. I., Rezek, Z., Lin, S. C., Alvarez-Fernández, E., Barshay-Szmidt, C. C., Hallett-Desguez, E., Reed, D., Reed, K., Richter, D., Steele, T. E., Skinner, A., Blackwell, B., Doronicheva, E. & El-Hajraoui, M. On the industrial attributions of the Aterian and Mousterian of the Maghreb. *J. Hum. Evol.* **64**, 194–210 (2013).

Douze, K. & Delagnes, A. The pattern of emergence of a Middle Stone Age tradition at Gademotta and Kulkuletti (Ethiopia) through convergent tool and point technologies. *J. Hum. Evol.* **91**, 93–121 (2016).

Douze, K. *et al.* A West African Middle Stone Age site dated to the beginning of MIS 5: archaeology, chronology, and paleoenvironment of the Ravin Blanc I (eastern Senegal). *J. Hum. Evol.* **154**, 102952 (2021).

Drake, N. A., Blench, R. M., Armitage, S. J., Bristow, C. S. & White, K. H. Ancient watercourses and biogeography of the Sahara explain the peopling of the desert. *Proc. Natl Acad. Sci. USA* **108**, 358–462 (2011).

Fusco, M., Carletti, E., Zerboni, A. & Gallinaro, M. Lithic variability and raw material exploitation at the Middle Stone Age (MSA) site of Gotera, southern Ethiopia: a combined technological and quantitative approach. *J. Lithic Stud.* **8**, 1–29 (2021).

Groos, A. R., Akçar, N., Yesilyurt, S., Miehe, G., Vockenhuber, C. & Viet, H. Non-uniform Late Pleistocene glacier fluctuations in tropical Eastern Africa. *Sci. Adv.* **7**, eabb6826 (2021).

Grun, R. Direct dating of Florisbad hominid. *J. Hum. Evol.* **43**, 27–32 (2016).

Hublin, J.-J. *et al.* New fossils from Jebel Irhoud, Morocco and the pan-African origin of Homo sapiens. *Nature* **546**, 289–292 (2017).

Jones, S. *et al.* Patterns of hominin occupation and cultural diversity across the Gebel Akhdar of Northern Libya over the last 200 kyr. In *Africa from MIS 6–2: Population Dynamics and Paleoenvironments* (Springer, 2016).

Leplongeon, A. The Palaeolithic peopling of North-Eastern Africa in its macroregional context. *Anthropology* **126**, 103015 (2022).

Martinón-Torres, M. *et al.* Earliest known human burial in Africa. *Nature* **593**, 95–100 (2021).

McDougall, I., Brown, F. & Fleagle, J. Stratigraphic placement and age of modern humans from Kibish, Ethiopia. *Nature***433**, 733–736 (2005).

McBrearty, S. & Brooks, A. S. The revolution that wasn’t: A new interpretation of the origin of modern human behaviour. *J. Hum. Evol.* **39**, 453–563 (2000).

Miller, J. M. & Wang, Y. V. Ostrich eggshell beads reveal 50,000-year-old social network in Africa. *Nature* **601**, 234–239 (2022).

Mirazón Lahr, M. & Foley, F. Human evolution in late Quaternary eastern Africa. In *Africa from MIS 6–2: Population Dynamics and Paleoenvironments* (Springer, 2016).

Mussi, M. *et al.* Garba III (Melka Kunture, Ethiopia): a MSA site with archaic Homo sapiens remains revisited. *Quat. Int.***343**, 28–39 (2014).

Richter, D. *et al.* The age of the hominin fossils from Jebel Irhoud, Morocco, and the origins of the Middle Stone Age. *Nature* **546**, 293–296 (2017).

Roberts, P. *et al.* Late Pleistocene to Holocene human palaeoecology in the tropical environments of coastal eastern Africa. *Palaeogeogr. Palaeoclimatol. Palaeoecol.* **537**, 109438 (2020).

Samawi, O. & Hallinan, E. More Than Surface Finds: Nubian Levallois Core Metric Variability in the Egyptian Western Desert. *J. Afr. Archaeol.* **21**, 1 (2023).

Scerri, E.M.L. The Aterian and its place in the North African Middle Stone Age. *Quat. Int.* **300**, 111–130 (2013).

Scerri, E. The North African Middle Stone Age and its place in recent human evolution. *Evol. Anthropol.* **26**, 119–135 (2017).

Scerri, E.M.L., Drake, N.A., Jennings, R. & Groucutt, H.S. Earliest evidence for the structure of Homo sapiens populations in Africa. *Quat. Sci. Rev.* **101**, 207–216 (2014). https://doi.org/10.1016/j.quascirev.2014.07.019.

Scerri, E.M.L. & Spinapolice, E.E. Lithics of the North African Middle Stone Age: Assumptions, evidence, and future directions. *J. Anthropol. Sci.* **97**, 1–36 (2019).

Scerri, E.M.L. & Will, M. The revolution that still isn’t: The origins of behavioural complexity in Homo sapiens. *J. Hum. Evol.* **179**, 103358 (2023).

Shea, J.J. The origins of lithic projectile point technology: Evidence from Africa, the Levant, and Europe. *J. Archaeol. Sci.* **33**, 823–846 (2006).

Shea, J.J. *Prehistoric Stone Tools of Eastern Africa: A Guide* (Cambridge University Press, 2020).

Shipton, C. et al. 78,000-year-old record of Middle and Later Stone Age innovation in an East African tropical forest. *Nat. Commun.* **9**, 28 (2018).

Timbrell, L. Ecology and demography of early Homo sapiens: A synthesis of archaeological and climatic data from eastern Africa. *Azania* **59**(1), 76–110 (2024).

Timbrell, L., Grove, M., Manica, A., Rucina, S. & Blinkhorn, J. A spatiotemporally explicit palaeoenvironmental framework for the Middle Stone Age of eastern Africa. *Sci. Rep.* **12**, 3689 (2022).

Tryon, C.A. The Middle/Later Stone Age transition and cultural dynamics of late Pleistocene East Africa. *Evol. Anthropol.* **28**, 267–282 (2019).

Tryon, C.A. & Faith, J.T. Variability in the Middle Stone Age of eastern Africa. *Curr. Anthropol.* **54**, 58 (2013).

Tryon, C.A., Crevecoeur, I., Faith, J.T., Ekshtain, R., Nivens, J., Patterson, D., Mbua, E.N. & Spoor, F. Late Pleistocene age and archaeological context for the hominin calvaria from GvJm-22 (Lukenya Hill, Kenya). *Proc. Natl Acad. Sci. USA***112**, 2682–2687 (2015).

Potts, R. et al. Environmental dynamics during the onset of the Middle Stone Age in eastern Africa. *Science* **360**, 86–90 (2018).

Potts, R. et al. Increased ecological resource variability during a critical transition in hominin evolution. *Nature* **6**, eabc8975 (2020).

Van Peer, P. et al. The early to middle Stone Age transition and the emergence of modern human behaviour at site 8-B-11 Sai Island, Sudan. *J. Hum. Evol.* **45**, 187–193 (2003).

White, T.D., Asfaw, B., DeGusta, D., Gilbert, H., Richards, G.D., Suwa, G. & Howell, F.C. Pleistocene Homo sapiens from Middle Awash, Ethiopia. *Nature* **433**, 742–747 (2003).

Wilkins, J. Is it time to retire NASTIES in Southern Africa? Moving beyond the culture-historical framework for Middle Stone Age lithic assemblage variability. *Lithic Technol.* **45**, 295–307 (2020).

Will, M., Mackay, A. & Phillips, N. Implications of Nubian-Like Core Reduction Systems in Southern Africa for the Identification of Early Modern Human Dispersals. *PLoS ONE* **10**(6), e0131824 (2015).
